# Stimulation of the beta-2-adrenergic receptor with salbutamol activates human brown adipose tissue

**DOI:** 10.1016/j.xcrm.2023.100942

**Published:** 2023-02-21

**Authors:** Maaike E. Straat, Carlijn A. Hoekx, Floris H.P. van Velden, Lenka M. Pereira Arias-Bouda, Lauralyne Dumont, Denis P. Blondin, Mariëtte R. Boon, Borja Martinez-Tellez, Patrick C.N. Rensen

**Affiliations:** 1 Division of Endocrinology, Department of Medicine, Leiden University Medical Center, Albinusdreef 2, 2333 ZA Leiden, the Netherlands; 2 Einthoven Laboratory for Experimental Vascular Medicine, Leiden University Medical Center, Albinusdreef 2, 2333 ZA Leiden, the Netherlands; 3 Section of Nuclear Medicine, Department of Radiology, Leiden University Medical Center, 2333 ZA Leiden, the Netherlands; 4 Centre de Recherche du Centre Hospitalier Universitaire de Sherbrooke, Sherbrooke, QC J1H 5N4, Canada; 5 Department of Physiology-Pharmacology, Université de Sherbrooke, Sherbrooke, QC J1H 5N4, Canada; 6 Department of Medicine, Division of Neurology, Université de Sherbrooke, Sherbrooke, QC J1H 5N4, Canada

**Keywords:** brown fat, cardiometabolic diseases, responders, energy expenditure, glucose metabolism, indirect calorimetry, lipoprotein metabolism, positron emission tomography, propranolol

## Abstract

While brown adipose tissue (BAT) is activated by the beta-3-adrenergic receptor (ADRB3) in rodents, in human brown adipocytes, the ADRB2 is dominantly present and responsible for noradrenergic activation. Therefore, we performed a randomized double-blinded crossover trial in young lean men to compare the effects of single intravenous bolus of the ADRB2 agonist salbutamol without and with the ADRB1/2 antagonist propranolol on glucose uptake by BAT, assessed by dynamic 2-[^18^F]fluoro-2-deoxy-D-glucose positron emission tomography-computed tomography scan (i.e., primary outcome). Salbutamol, compared with salbutamol with propranolol, increases glucose uptake by BAT, without affecting the glucose uptake by skeletal muscle and white adipose tissue. The salbutamol-induced glucose uptake by BAT positively associates with the increase in energy expenditure. Notably, participants with high salbutamol-induced glucose uptake by BAT have lower body fat mass, waist-hip ratio, and serum LDL-cholesterol concentration. In conclusion, specific ADRB2 agonism activates human BAT, which warrants investigation of ADRB2 activation in long-term studies (EudraCT: 2020-004059-34).

## Highlights

• ADRB2 agonism by salbutamol increases glucose uptake by human BAT *in vivo*
• ADRB2 agonism increases energy expenditure correlating with BAT glucose uptake
• ADRB2 agonism does not increase glucose uptake by skeletal muscle and WAT
• ADRB1/2 antagonism blocks salbutamol-induced glucose uptake by human BAT

## Graphical abstract

**Figure:**
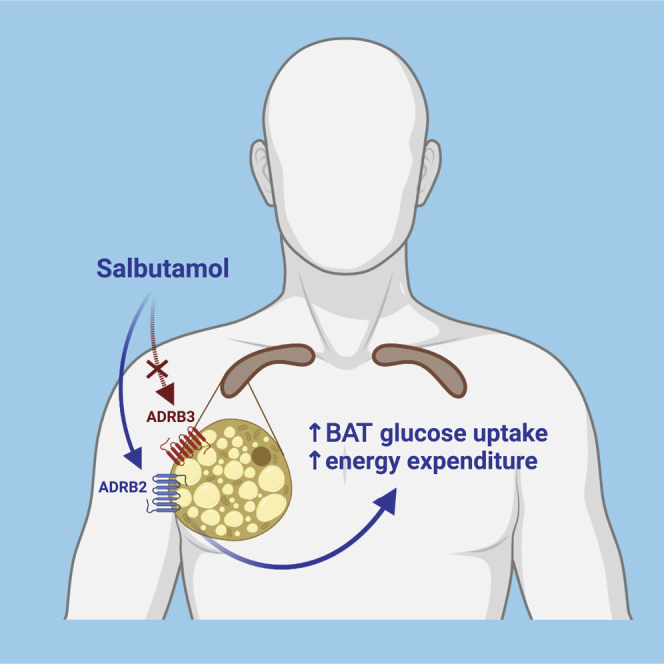

## Teaser

Brown adipose tissue (BAT) activation is an attractive strategy to improve cardiometabolic health. While ADRB3 is responsible for BAT activation in rodents, the ADRB2 predominates on human brown adipocytes. Straat et al. reveal that ADRB2 agonism by salbutamol increases glucose uptake by human BAT and energy expenditure *in vivo*.

## Introduction

Over the last decades, brown adipose tissue (BAT) has become an attractive target to stimulate energy dissipation to improve cardiometabolic health.^1^ BAT is a highly vascularized thermogenic organ mainly located in the deep neck region, along large blood vessels, and in the supraclavicular area, and it combusts triglyceride-derived fatty acids and glucose into heat.^2^^,^^3^^,^^4^ Naturally, the most potent activator of BAT is cold exposure, which increases sympathetic outflow toward beta-adrenergic receptors on BAT.^5^^,^^6^

In rodents, the beta-3-adrenergic receptor (ADRB3) is the main adrenergic receptor found on brown adipocytes, and activation of the ADRB3 has been shown to effectively activate BAT and improve cardiometabolic outcomes in mice.^7^^,^^8^^,^^9^ In humans, however, the involvement of ADRB3 in BAT activation is less clear.^10^^,^^11^^,^^12^^,^^13^^,^^14^^,^^15^^,^^16^ The ADRB3 agonist mirabegron increases the uptake of the glucose analog 2-[^18^F]fluoro-2-deoxy-D-glucose ([^18^F]FDG) by BAT, increases whole-body lipolysis, and increases resting energy expenditure. Nevertheless, this only occurs after administration of a supratherapeutic dose of 200 mg,^10^^,^^11^^,^^12^ which highly exceeds the therapeutic dose of 50 mg to treat hyperactive bladder in the clinic. In addition, at 200 mg, cardiovascular side effects occur, such as an increase in heart rate and systolic blood pressure, raising the possibility that mirabegron cross-reacts with other beta-adrenergic receptors such as the ADRB1 and ADRB2 that are also present in the cardiovascular system and as such contribute to the increase in energy expenditure.^17^

Indeed, we recently showed that the therapeutic dose of 50 mg mirabegron is ineffective to increase oxidative metabolism in BAT, and that the ADRB2 is in fact the dominant adrenergic receptor expressed in human BAT biopsies and brown adipocytes, while the expression of ADRB3 is negligible.^12^ Accordingly, evidence supporting the hypothesis that ADRB2 is responsible for stimulating thermogenesis in human BAT stems from our *in vitro* experiments in human brown adipocytes, where we demonstrated that (1) stimulation with mirabegron did not increase oxygen consumption; (2) stimulation with the ADRB2 agonist formoterol increased oxygen consumption, which was inhibited when pre-exposed with a selective ADRB2 antagonist; and (3) knockdown of ADRB2, but not ADRB1 or ADRB3, reduced norepinephrine-stimulated oxygen consumption.^12^ Therefore, the aim of this study was to investigate the role of ADRB2 in activation of human BAT *in vivo*. To this end, as a proof of concept, we evaluated the acute effect of the specific ADRB2 agonist salbutamol on glucose uptake by BAT without and with the ADRB1/2 antagonist propranolol in healthy, lean men.

## Results

### Salbutamol increases heart rate and tends to increase energy expenditure

**Figure 1:**
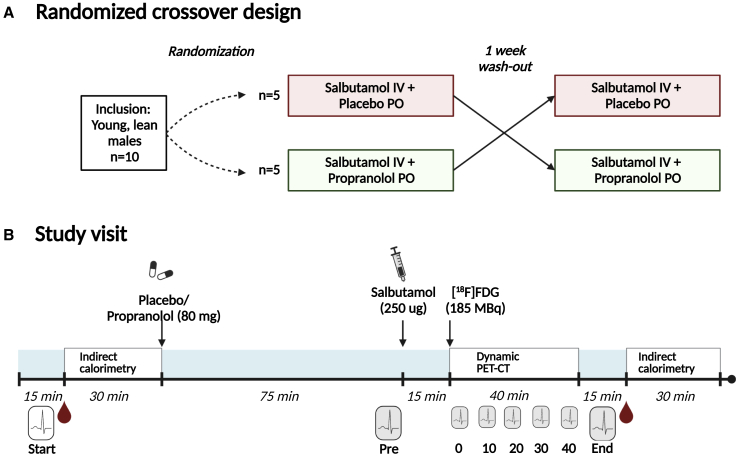
Study design and timeline of study procedures (A) This study had a randomized, double-blinded, crossover design. (B) Both study visits started with the measurement of blood pressure and heart rate (indicated by the ECG icon). Thereafter, the first blood sample (indicated by blood drop icon) was drawn, followed by an indirect calorimetry measurement for 30 min. Then, participants received either placebo or propranolol (80 mg, in two capsules; per oral, PO), depending on the study visit. After 75 min, blood pressure and heart rate were measured again, and a single bolus of salbutamol (250 μg; intravenous, IV) was injected over a continuous time course of 5 min. 15 min after initiation of the injection, a low-dose computed tomography (CT) scan was performed, directly followed by injection of 2-[^18^F]fluoro-2-deoxy-D-glucose ([^18^F]FDG; 185 MBq) and a dynamic positron emission tomography (PET) acquisition, during which heart rate was monitored. After termination of the scan, blood pressure and heart rate were measured, the final blood sample was drawn, and indirect calorimetry was performed for 30 min.

**Figure 2:**
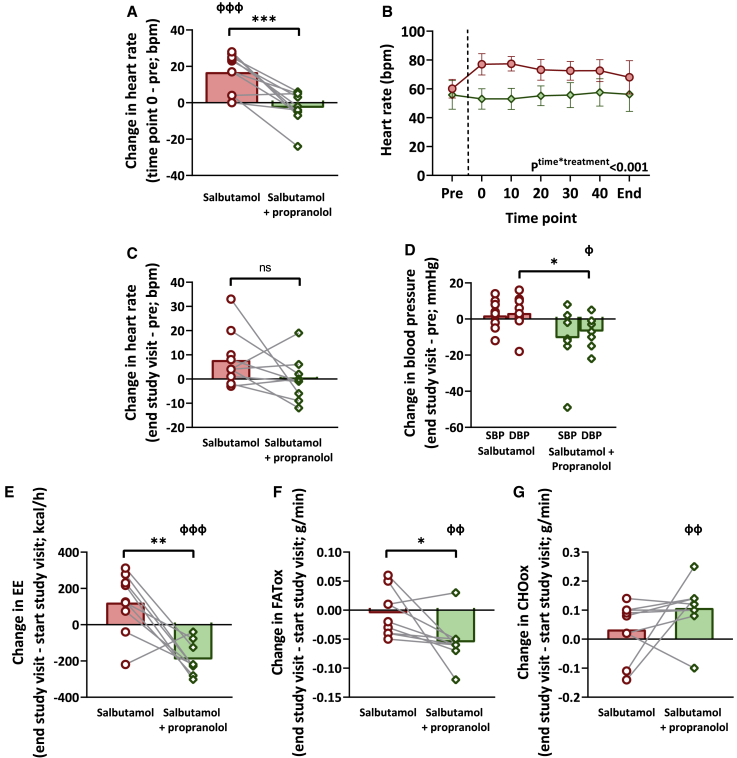
The effect of salbutamol vs. salbutamol with propranolol on heart rate, blood pressure, energy expenditure, and nutrient oxidation rates (A, C–G)The direct change in heart rate (n = 10) (A) and change over the study day of heart rate (n = 9) (C), systolic blood pressure (SBP) (n = 10) and diastolic blood pressure (DBP) (n = 9) (D), energy expenditure (EE) (n = 9) (E), fatty acid oxidation (FATox) (n = 9) (F), and carbohydrate oxidation (CHOox) (n = 9) (G) after salbutamol (red bars with circles) vs. salbutamol with propranolol (green bars with diamonds). For one participant, EE measurement failed due to technical issues. In one participant, a measurement of heart rate at the end of the study after salbutamol with propranolol is missing. General linear models with repeated measures and pairwise comparisons were used to test the effect of treatment and to compare the treatment regimens. Bars represent means, circles/diamonds represent individual values, and gray lines represent paired data. Before vs. after treatment: ^ф^p ≤ 0.05, ^фф^p ≤ 0.01, ^ффф^p ≤ 0.001. Salbutamol vs. salbutamol with propranolol: ∗p ≤ 0.05, ∗∗p ≤ 0.01, ∗∗∗p ≤ 0.001. (B) The effect of salbutamol (n = 10; red circles) vs. salbutamol with propranolol (n = 9; green diamonds) on heart rate over time. Vertical dashed line represents the moment of the administration of salbutamol. General linear model with repeated measures was used to test for an interaction between treatment regime and the effect of treatment over time. Bars represent means, and error bars represent SD.

In total, 10 young (age: 24.4 ± 4.3 years) and lean (body mass index: 23.1 ± 2.3 kg/m^2^) males were included in this study and participated in two experimental study visits (see Figures 1A and 1B). A single intravenous bolus of salbutamol (250 μg) acutely increased heart rate (+16.9 ± 10.5 bpm, p = 0.001), but not when combined with propranolol (−2.8 ± 8.9 bpm, p = 0.35; interaction between treatments p < 0.001; Figure 2A). This initial effect of salbutamol injection on heart rate gradually faded (Figure 2B), resulting in a non-significant difference between treatments at the end of the study visit (Figure 2C). Salbutamol did not affect systolic blood pressure (+2.4 ± 8.0 mm Hg; p = 0.38) or diastolic blood pressure (+3.2 ± 9.7 mm Hg, p = 0.35) (i.e., end of treatment visit vs. before treatment). Salbutamol combined with propranolol tended to decrease systolic blood pressure (−10.7 ± 16.5 mm Hg, p = 0.09) and decreased diastolic blood pressure (−7.0 ± 8.0 mm Hg, p = 0.03) (i.e., end of treatment visit vs. before treatment) (Figure 2D). It should be noted that blood pressure could not be assessed in between these measurements, and initial effects of salbutamol on systolic and diastolic blood pressure may have been missed.

Next, we assessed the effect of salbutamol on energy expenditure and substrate utilization. For one male, the gas exchange measurement failed due to technical issues, leaving a total of nine males for these analyses. Salbutamol tended to increase energy expenditure (+7.2%, +122 ± 168 kcal/day, p = 0.06), whereas salbutamol with propranolol decreased energy expenditure (−9.4%, −192 ± 91 kcal/day, p < 0.001), leading to a significantly different change in energy expenditure between the two treatment regimens (p = 0.005; Figure 2E). Of note, the salbutamol-induced percentual change in energy expenditure did not correlate with the increase in heart rate, both parameters defined as the change between “end study visit” *minus* “start study visit” (Spearman’s rho: −0.59, p = 0.10; Figure S1). Fat oxidation did not change after salbutamol (−0.004 ± 0.04 g/min, p = 0.77), but it decreased after salbutamol with propranolol (−0.06 ± 0.04 g/min, p = 0.003; change between treatments p = 0.03; Figure 2F). Carbohydrate oxidation did not change after salbutamol (+0.03 ± 0.10 g/min, p = 0.34), but it increased after salbutamol with propranolol (+0.12 ± 0.09 g/min, p = 0.009; change between treatments: p = 0.19; Figure 2G).

### Salbutamol increases net glucose uptake by brown adipose tissue, and this positively correlates to the change in energy expenditure

**Figure 3:**
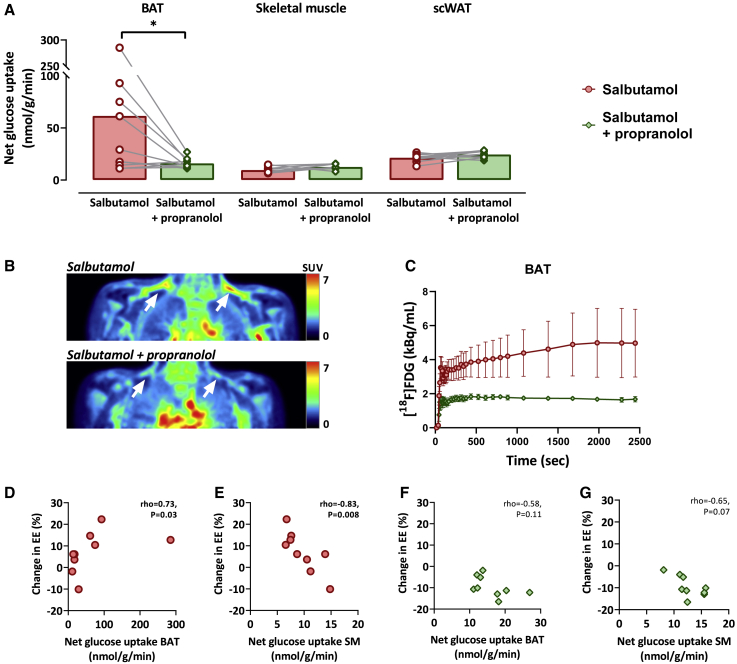
The effect of salbutamol vs. salbutamol with propranolol on glucose uptake by brown adipose tissue, skeletal muscle, and subcutaneous white adipose tissue and the association with the change in energy expenditure (A) The glucose uptake by human brown adipose tissue (BAT), skeletal muscle (i.e., average of m. pectoralis, m. trapezius, m. deltoideus, and m. sternocleidomastoideus), and subcutaneous white adipose tissue (scWAT) after salbutamol (n = 10) vs. salbutamol with propranolol (n = 10). A paired Student’s t test, or nonparametric equivalent, was used to compare the two treatment regimes. Bars represent means, dots/diamonds represent individual values, and gray lines represent paired data. ∗p ≤ 0.05. (B) Positron emission tomography images of the supraclavicular area illustrating the [^18^F]fluorodeoxyglucose [^18^F]FDG uptake, expressed by body-weighted standardized uptake values (SUVs), in response to salbutamol (top) and salbutamol with propranolol (bottom). The same representative participant is presented for both images. White arrows show supraclavicular BAT depots. (C) Time-activity curve showing the concentration of [^18^F]FDG in BAT depots. Left and right, and all participants (n = 10) are averaged. Data represent mean with SEM. (D and E) Correlation plots between the change in energy expenditure (EE) (%) and the glucose uptake by human BAT after salbutamol (D) and skeletal muscle (SM) after salbutamol (E) (n = 9). For one participant, EE measurement failed due to technical issues. (F and G) Correlation plots between the change in EE and the glucose uptake by human BAT after salbutamol with propranolol (F) and SM after salbutamol with propranolol (G) (n = 9).

**Figure 4:**
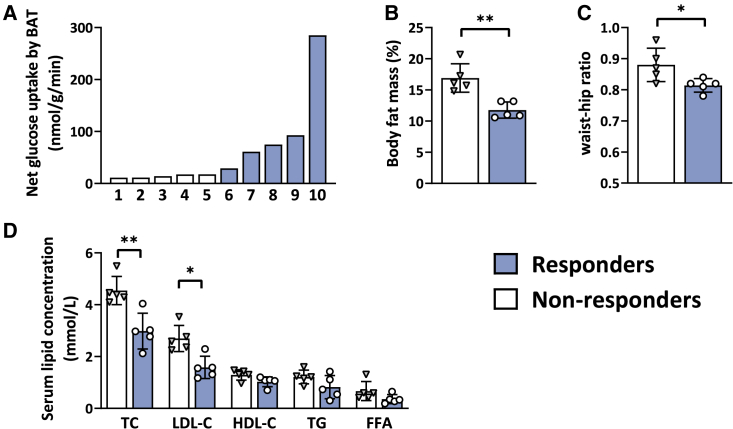
Differences in body composition and baseline serum lipid concentrations between non-responders and responders in terms of salbutamol-induced glucose uptake by brown adipose tissue (A) Waterfall plot showing the distribution in net glucose uptake by supraclavicular brown adipose tissue (BAT; left and right averaged). Each bar represents the individual value of a participant. (B–D) Differences in body fat mass percentage (B), waist-hip ratio (C), and baseline serum concentrations of total cholesterol (TC), low-density lipoprotein cholesterol (LDL-C), high-density lipoprotein cholesterol (HDL-C), triglycerides (TG), and free fatty acids (FFA) (D) between participants who showed a high salbutamol-induced net glucose uptake by brown adipose tissue (“responders,” blue bars with circles; n = 5) vs. participants who showed low salbutamol-induced net glucose uptake by brown adipose tissue (“non-responders,” white bars with triangles; n = 5). Values illustrated in the figures were measured at baseline during the placebo-visit. A paired Student’s t test, or nonparametric equivalent, was used to compare the two groups. Bars represent means, and error bars represent SD. ∗p ≤ 0.05, ∗∗p ≤ 0.01.

We next assessed the effect of the ADRB2 agonist salbutamol on net glucose uptake by supraclavicular BAT, as calculated from the [^18^F]FDG influx rate. Salbutamol, compared with salbutamol with propranolol, increased the net glucose uptake by BAT (salbutamol vs. salbutamol with propranolol: 67.1 ± 87.0 nmol/g/min vs. 16.2 ± 5.2 nmol/g/min, p = 0.03; Figures 3A–3C). In contrast, after salbutamol, glucose uptake by skeletal muscle (9.5 ± 3.0 nmol/g/min vs. 12.6 ± 2.4 nmol/g/min, p = 0.06) and subcutaneous white adipose tissue (scWAT) (21.4 ± 3.7 nmol/g/min vs. 24.5 ± 3.4 nmol/g/min, p = 0.06; Figure 3A) tended to be lower compared with after salbutamol with propranolol. Interestingly, we observed a physiologically plausible outlier with very high glucose uptake values by BAT (285.1 nmol/g/min, see identification number 10 in Figures 4A and S2). After sensitivity analyses excluding the values of this participant, differences in glucose uptake by BAT after salbutamol vs. salbutamol with propranolol remained significant (salbutamol vs. salbutamol with propranolol: 36.7 ± 31.2 nmol/g/min vs. 14.9 ± 3.2 nmol/g/min, p = 0.05; data not shown). BAT volumes were not calculated as these are markedly influenced by various factors (e.g., diet, intracellular triglyceride stores, and thresholds for standard uptake value).^18^^,^^19^

After salbutamol, glucose uptake by BAT was positively associated with the percentage change in energy expenditure (Spearman’s rho = 0.73, p = 0.03; Figure 3D), whereas the glucose uptake by skeletal muscle was negatively associated (Spearman’s rho = −0.83, p = 0.008; Figure 3E). After salbutamol with propranolol, no significant correlation was found between the glucose uptake by BAT and energy expenditure (Spearman’s rho = −0.58, p = 0.11; Figure 3F) and a tendency between the change in glucose uptake by skeletal muscle and energy expenditure (Spearman’s rho = −0.65, p = 0.07; Figure 3G). In addition, we observed a tendency between a delta of energy expenditure (EE) (i.e., the change of EE after 45 min of salbutamol injection *minus* the change of EE after salbutamol with propranolol) with the delta of net glucose uptake by BAT (i.e., the value of BAT after 45 min of salbutamol injection *minus* the value of BAT after salbutamol with propranolol) (Spearman’s rho = 0.70, p = 0.05; Figure S3A) and a negative correlation with the delta of net glucose uptake by skeletal muscle (SM) (i.e., the value of SM after 45 min of salbutamol injection minus the value of SM after salbutamol with propranolol (Spearman’s rho = −0.73, p = 0.025 Figure S3B). Furthermore, during both treatment regimes, no significant correlations were found between the change in glucose uptake by scWAT and EE (salbutamol: Spearman’s rho = −0.16, p = 0.68; salbutamol with propranolol: Spearman’s rho = −0.32, p = 0.41; not shown).

### High responders to salbutamol-induced glucose uptake by BAT have a more beneficial metabolic phenotype than low responders

A marked variability was observed between participants in the salbutamol-induced glucose uptake by BAT. Specifically, as is evident from Figures 4A and S2, five participants showed a high glucose uptake by BAT (ranging from 29.2 to 285.1 nmol/g/min; “responders”), whereas the other five participants showed low glucose uptake (ranging from 11.1 to 17.6 nmol/g/min; “non-responders”; p = 0.008). Compared with non-responders, responders had a lower body fat mass (11.8% ± 1.3% vs. 16.9% ± 2.3%, p = 0.008; Figure 4B), lower waist-hip ratio (WHR) (0.8 ± 0.02 vs. 0.9 ± 0.1, p = 0.03; Figure 4C), and lower serum concentrations of total cholesterol (3.0 ± 0.7 mmol/L vs. 4.5 ± 0.5 mmol/L, p = 0.008; Figure 4D) and LDL-cholesterol (1.6 ± 0.4 mmol/L vs. 2.7 ± 0.5 mmol/L, p = 0.02; Figure 4D). No significant differences were observed in glucose, insulin, or C-peptide levels between non-responders and responders (all p ≥ 0.1, see Table S1). Moreover, responders tended to have a higher heart rate at the start of the study visit (77.8 ± 10.0 bpm vs. 66.5 ± 8.3 bmp, p = 0.06), without differences in baseline EE (1,887 ± 384 kcal/day vs. 2,056 ± 68 kcal/day, p = 1.0). There was no significant difference between the groups in salbutamol-induced change in EE (+10.0% ± 12.1% vs. +3.5% ± 3.8%, p = 0.35) or heart rate (+12.2 ± 13.2 bpm vs. +21.6 ± 4.4 bpm, p = 0.55). A full overview of baseline characteristics between the two phenotypes can be found in Table S1.

### Salbutamol does not affect serum lipid or glucose concentrations

**Figure 5:**
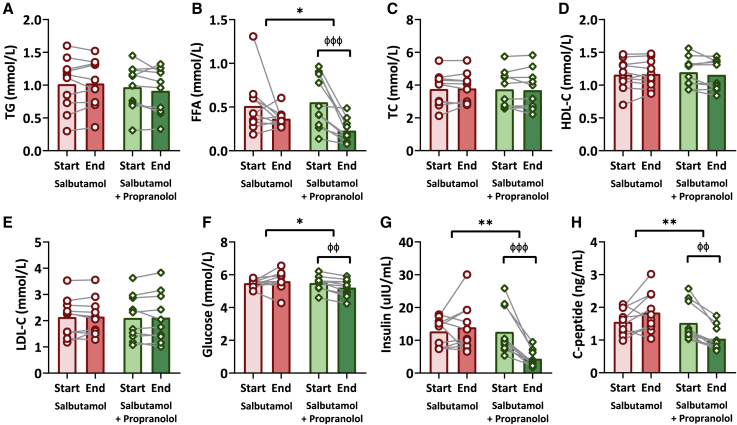
The effect of salbutamol vs. salbutamol with propranolol on serum concentrations of lipid and glucose metabolism The effect of salbutamol (red bars with circles; n = 10) vs. salbutamol with propranolol (green bars with diamonds; n = 10) on serum concentrations of triglycerides (TG; A), free fatty acids (FFA; B), total cholesterol (TC; C), high-density lipoprotein cholesterol (HDL-C; D), low-density lipoprotein cholesterol (LDL-C, E), glucose (F), insulin (G), and C-peptide (H). General linear models with repeated measures and pairwise comparisons were used to test the effect of treatment and to compare the treatment regimens. Bars represent means, dots/diamonds represent individual values, and gray lines represent the paired nature of the data. Start vs. end: ^фф^p ≤ 0.01, ^ффф^p ≤ 0.001. Salbutamol vs. salbutamol with propranolol: ∗p ≤ 0.05, ∗∗p ≤ 0.01.

Lastly, we aimed to assess the acute effects of salbutamol on measures of lipoprotein and glucose metabolism. Salbutamol did not affect serum concentrations of triglycerides (start vs. end study day: 1.0 ± 0.4 mmol/L vs. 1.0 ± 0.4 mmol/L, p = 0.78), free fatty acids (FFA) (0.5 ± 0.3 mmol/L vs. 0.4 ± 0.1 mmol/L, p = 0.21), total cholesterol (3.8 ± 1.0 mmol/L vs. 3.8 ± 0.9 mmol/L, p = 0.74), HDL-cholesterol (1.2 ± 0.2 mmol/L vs. 1.2 ± 0.2 mmol/L, p = 0.70), or LDL-cholesterol (2.1 ± 0.7 mmol/L vs. 2.2 ± 0.7 mmol/L, p = 0.78; Figures 5A–5E). Salbutamol with propranolol only decreased serum FFA levels (0.6 ± 0.3 mmol/L vs. 0.2 ± 0.1 mmol/L, p < 0.001; Figure 5B). Moreover, salbutamol did not affect serum concentrations of glucose (5.5 ± 0.2 mmol/L vs. 5.6 ± 0.6 mmol/L, p = 0.45), insulin (12.7 ± 4.3 μU/mL vs. 14.0 ± 7.3 μU/mL, p = 0.75), or C-peptide (1.6 ± 0.4 ng/mL vs. 1.8 ± 0.6 ng/mL, p = 0.08), whereas salbutamol with propranolol decreased serum concentrations of glucose (5.5 ± 0.4 mmol/L vs. 5.2 ± 0.5 mmol/L, p = 0.002), insulin (12.6 ± 7.1 μU/mL vs. 4.4 ± 2.5 μU/mL, p < 0.001), and C-peptide (1.5 ± 0.6 ng/mL vs. 1.0 ± 0.4 ng/mL, p = 0.001; Figures 5F–5H). Importantly, the glucose uptake by BAT after salbutamol with propranolol was not associated with the decrease in glucose (Spearman’s rho = 0.28, p = 0.43) nor insulin (Spearman’s rho = 0.45, p = 0.19; not shown).

## Discussion

In this proof-of-concept study, we show that pharmacological stimulation of the ADRB2 acutely increases glucose uptake by human BAT *in vivo*. Specifically, we demonstrate that a single intravenous administration of the specific ADRB2 agonist salbutamol increased glucose uptake by BAT, which could be largely prevented when blocking the ADRB1/2 with propranolol. The salbutamol-induced uptake of glucose by BAT, but not SM, was positively associated with whole-body EE. Notably, participants with high salbutamol-induced glucose uptake by BAT had a more favorable metabolic phenotype (among other lower WHR and LDL-cholesterol levels) compared with participants with low glucose uptake by BAT. Together, our data underline the relevance of the ADRB2 for sympathetically induced glucose uptake by human BAT.

By revealing that stimulation of the ADRB2 increases glucose uptake by human BAT, we now provide *in vivo* evidence for our recent findings that the ADRB2 is the adrenergic receptor that activates human brown adipocytes *in vitro*.^12^ Moreover, since blocking the ADRB1/2 effectively inhibited the salbutamol-induced glucose uptake by BAT in our study, the ADRB3 apparently was not involved. Over the last decades, many research groups have focused on mimicking the sympathetic activation of BAT by targeting the ADRB3.^10^^,^^13^^,^^14^^,^^20^ Direct targeting the ADRB3 using CL 316243, one of the most commonly used ADRB3 agonists, effectively activates BAT and improves cardiometabolic outcomes in mice.^7^^,^^9^ However, pharmacological targeting of the ADRB3 by the use of mirabegron led to inconsistent results in humans.^10^^,^^11^^,^^12^^,^^13^^,^^14^^,^^15^^,^^16^ In fact, mirabegron only activates human BAT oxidative metabolism at maximal allowable dose (i.e., 200 mg), which also induces cardiovascular side effects,^11^^,^^12^^,^^21^ suggesting cross-activation with other beta-adrenergic receptors. Indeed, the human heart mainly expresses ADRB1 (80%) and ADRB2 (20%), with negligible expression of ADRB3.^22^ In human BAT, in principle all three receptors are present.^12^^,^^23^^,^^24^^,^^25^^,^^26^ In immortalized brown adipocytes originating from just a single donor, stimulation of the ADRB1, but not ADRB2 or ADRB3, increased uncoupling protein 1 expression and lipolysis.^26^ In contrast, we demonstrated using both immortalized and *in vitro* differentiated primary brown adipocytes from multiple donors from independent labs that ADRB2 agonism increases respiration, and knockdown of the ADRB2, but not ADRB1 and ADRB3, hampers norepinephrine-induced respiration.^12^ Taken together, although the involvement of the ADRB1 cannot be excluded, the ADRB2 seems to play the most important role in sympathetic activation of human BAT *in vivo*.

We show that ADRB2 stimulation with salbutamol tended to increase whole-body EE, and the change in EE correlated positively with the net glucose uptake by BAT. This is in line with previous studies that also demonstrated that intravenous administration of salbutamol with and without atenolol (i.e., ADRB1 blocker) increased EE, although in those studies the tissues involved had not been explored.^27^^,^^28^^,^^29^ Besides BAT, SMs are also considered responsible for the increase in thermogenesis during cold exposure, and deeper and more centrally located SMs have been shown to contribute to the cold-induced glucose turnover.^30^ However, we here show that the change in EE after salbutamol in fact negatively correlates with the net glucose uptake by SM. Moreover, comparable to previous findings that acute ADRB2 stimulation does not affect insulin-stimulated glucose uptake by SM, we did not find an increase in glucose uptake by SM after acute salbutamol administration. Interestingly, long-term (4 weeks) daily inhalation of terbutaline (i.e., an ADRB2 agonist) increases insulin-stimulated whole-body glucose disposal, without changes in GLUT4 in SM or abdominal WAT.^31^ Although it has been shown that this may in part be due to muscle hypertrophy,^31^ we propose a potential role for BAT through increased glucose uptake coinciding with increased energy dissipation.

Previous studies demonstrated that continuous or repetitive intravenous administration of salbutamol with and without atenolol increases lipolysis and fat oxidation together with an increase in EE.^27^^,^^28^^,^^29^ In the present study, we did not observe a change in circulating lipids or in fat oxidation after a single bolus of salbutamol. Importantly, we performed indirect calorimetry and blood sampling approximately 70 min after salbutamol administration. Hence, we may have missed an acute effect of salbutamol on lipolysis and fat oxidation. Indeed, salbutamol with atenolol (i.e., resulting in specific ADRB2 stimulation) increases fat oxidation only after 45 min of continuous intravenous administration.^27^ Furthermore, we used a single intravenous bolus of salbutamol, which may have not been sufficiently potent to induce a lipolytic effect in white adipose tissue. Interestingly, it has been reported previously that the salbutamol-induced increase in EE is independent of circulating fatty acid levels, as substrate utilization switches from fat to carbohydrate when peripheral lipolysis is blocked with acipimox (i.e., a niacin derivative that inhibits adipose triglyceride lipase).^28^ It is conceivable that the single bolus of salbutamol only transiently increases lipolysis, circulating fatty acid levels, and fat oxidation, and it eventually increases EE due to an increase in both fat and glucose oxidation.

The finding that a single bolus of salbutamol activates human BAT and whole-body energy metabolism is a promising lead for the development of BAT-targeted drugs to treat obesity-related cardiometabolic disorders. Nevertheless, although salbutamol is approved in the clinic for the treatment of respiratory diseases, it is unlikely that conventional tissue-unspecific ADRB2 agonism will be applied to treat cardiometabolic pathophysiology because of common cardiovascular side effects (i.e., increase in heart rate, blood pressure, and tremors). Moreover, pharmacological stimulation of the ADRB2 using salbutamol does not fully mimic the sympathetic cold-induced activation of BAT just yet, as during cold exposure heart rate decreases,^12^ possibly explained by cold-induced local norepinephrine release affecting tissue-specific beta- as well as alpha-receptors. Nevertheless, it is conceivable that BAT-targeted drug delivery systems may be developed. Such delivery systems have already been developed to target the liver, taking advantage of the exclusive expression of the asialoglycoprotein receptor on hepatocytes, and hepatocyte-targeted modulation of PCSK9 by Inclirisan has recently been approved by the FDA to reduce cardiovascular disease risk.^32^ Of note, a recent study indeed showed the feasibility of targeting adipose tissue using an adipose homing peptide.^33^

Interestingly, we found notable individual variability in the glucose uptake by BAT after salbutamol administration, with responders having a lower body fat mass percentage and lower WHR as well as lower total cholesterol and LDL-cholesterol levels compared with non-responders. This seems consistent with previous studies showing that [^18^F]FDG uptake by cold-exposed BAT^34^^,^^35^ or thermoneutral BAT^36^^,^^37^ is associated with a lower body fat mass percentage and/or less central adiposity. As an explanation, a higher body fat mass may influence the detectability of BAT using [^18^F]FDG positron emission tomography (PET) acquisitions, as human BAT is scattered between white adipocytes, and the proportion between classical BAT, beige/brite, and white adipose tissue varies substantially between individuals.^38^^,^^39^ Also, relatively high circulating triglyceride levels at baseline may interfere with glucose uptake by BAT. Under stimulated conditions, BAT increases oxidation of intracellular triglyceride-derived fatty acids, followed by replenishment of the intracellular lipid stores via the uptake of glucose and triglyceride-derived fatty acids from the circulation.^40^ A higher triglyceride-derived fatty acid flux toward the BAT will thus reduce its glucose uptake.^41^ However, in the current study, baseline triglyceride levels were not significantly different. Alternatively, variations in responsiveness of the ADRB2, due to gene polymorphisms, receptor sensitivity, and/or receptor density, could explain differences in salbutamol-induced glucose uptake by BAT and disparities in metabolic phenotype. Various polymorphisms in the ADRB2 gene have been identified, of which the two most commonly studied gene polymorphisms have a frequency of approximately 35% to 50% in Europe.^42^ Indeed, variations in polymorphisms of the ADRB2 gene can result in different thermogenic responses upon ADRB2 stimulation.^29^ In addition, loss-of-function mutations of the ADRB2 gene are related to higher body fat and circulating lipid variability,^43^^,^^44^^,^^45^^,^^46^^,^^47^^,^^48^^,^^49^ and low ADRB2 sensitivity and low ADRB2 density are linked to higher circulating triglycerides and LDL-cholesterol, respectively.^50^^,^^51^ With respect to the current study, the latter would suggest that the responsiveness of the ADRB2 influences both the metabolic phenotype as the salbutamol-induced glucose uptake by BAT, which may be an important factor to consider when targeting BAT to improve cardiometabolic health.

### Conclusions

In conclusion, we provide evidence that stimulation of the ADRB2 using salbutamol acutely increases the rate of glucose uptake by human BAT *in vivo*, which is suppressed after blocking the ADRB1/2, suggesting that this effect is not mediated by cross-reaction of salbutamol with the ADRB3. As such, these findings provide *in vivo* evidence for our recent findings that the ADRB2 is responsible for adrenergic stimulation of human brown adipocytes.^12^ Identification of ADRB2 as the most important receptor involved in the sympathetic activation of human BAT thermogenesis allows us to maximize the therapeutic potential of targeting BAT to combat cardiometabolic diseases.

### Limitations of the study

This study is not without limitations. While we used [^18^F]FDG as tracer for BAT activation, triglyceride-derived fatty acids are probably much more important as fuel for BAT oxidative metabolism.^40^ Nonetheless, a PET-compatible triglyceride tracer has not been described as yet. Alternative tracers include 14(R,S)-[^18^F]fluoro-6-thia-heptadecanoic acid or [^11^C]acetate, which trace circulating fatty acid uptake by BAT and oxidative metabolism and perfusion of BAT, respectively. Using these tracers, it has been reported that BAT glucose uptake can be uncoupled from BAT oxidative metabolism, as shown in individuals with obesity or type 2 diabetes who have maintained BAT oxidative metabolism and fatty acid uptake, despite reduced glucose uptake.^52^ Hence, future work should examine how the salbutamol-induced changes in BAT glucose uptake reflect changes in BAT oxidative metabolism. Furthermore, in the current study, we compared salbutamol treatment with salbutamol combined with propranolol. The addition of a third, vehicle-treated control condition would have been ideal. However, the [^18^F]FDG positron emission tomography-computed tomography (PET-CT) scans that are required to assess BAT activity in a reliable way are subjected to a high radiation burden (i.e., 4.2 mSv per scan). As the total annual radiation burden is not allowed to exceed 10 mSv in the Netherlands, we were unfortunately restricted to a maximum of two [^18^F]FDG PET-CT scans per person.^53^ One of our main research objectives was to eliminate the possibility that the stimulatory effect of salbutamol on glucose uptake by BAT is mediated via the ADRB3. Hence, we reasoned that we would gain the most scientific knowledge by studying the effects of salbutamol without and with the ADRB1/2 antagonist propranolol. Of note, a previous study showed similar rates of glucose uptake by BAT when participants were exposed to room temperature as we found in our study when participants were treated with salbutamol and propranolol, suggesting that the stimulatory effect of salbutamol was indeed effectively inhibited by propranolol to values that would have been observed under non-stimulated conditions.^54^ Another general limitation is that with [^18^F]FDG PET-CT analysis reliable measures of BAT volume cannot be obtained, as accumulation of [^18^F]FDG in BAT and therefore the volume of BAT that meets the arbitrary threshold used to define BAT are dependent upon (1) amount of tracer administered; (2) time between tracer administration and image acquisition; (3) whether the tissue activity is normalized to body weight or lean body mass; (4) and the type of PET scan used (each manufacturer and/or model from same manufacturer will have different sensitivities). A final limitation is that we could not measure the dynamic responses of blood pressure, EE, and nutrient oxidation rates during the PET image acquisition as we did for heart rate. Finally, only young, lean males were included in this study. As we already observe baseline differences between responders and non-responders considering body composition and circulating lipid levels, future studies should focus on metabolically compromised individuals and additionally include women.

## STAR★Methods

### Key resources table

**Table:** 

REAGENT or RESOURCE	SOURCE	IDENTIFIER
**Critical commercial assays**		

Free fatty acids Standard	Wako chemicals	270–7700
Reagent 1	Wako Chemicals	434–91795
Reagent 2	Wako Chemicals	436–91995
Triglycerides Standard	Roche Diagnostics	10,166,588
Reagent	Roche Diagnostics	11,489,232,216
Total Cholesterol	Roche Diagnostics	11,489,232,216
Standard	Instruchemie	1016
High-density Lipoprotein Cholesterol Reagent	Roche Diagnostics	11,489,232,216
Glucose human Reagent	Instruchemie	10,786
Standard	Sigma-Aldrich	G6918
Insulin	Meso Scale Diagnostics	K151S5-1
C-peptide	Meso Scale Diagnostics	K151X5D-1

**Software and algorithms**		

PMOD software	PMOD technologies	htttps://pmod.com; RRID:SCR_016547
Graphpad Prism 9	GraphPad Software	https://graphpad.com; RRID:SCR_002798
BioRender	BioRender	https://biorender.com; RRID:SCR_018361
SPSS	IBM Corporation	https://ibm.com/nl-en/products/spss-statistics; RRID:SCR_019096

**Other**		

2-[18F]fluoro-2-deoxy-D-glucose ([18F]FDG)	Curium Pharma	https://www.curiumpharma.com/nl/

### Resource availability

#### Lead contact

Further information and requests for resources and reagents should be directed to and will be fulfilled by the lead contact, Prof. Dr. Patrick C.N. Rensen (p.c.n.rensen@lumc.nl).

#### Materials availability

This study did not generate new unique reagents.

### Experimental model and subject details

#### Study design

We performed a single center randomized double-blinded crossover trial to assess whether ADRB2 agonism activates human brown adipose tissue (BAT). The intervention consisted of a single intravenous bolus of salbutamol (250 μg) in combination with orally administered propranolol (80 mg) or placebo, in random order after a seven-day wash-out period (Figure 1A). On both study days all participants underwent a dynamic [^18^F]FDG Positron Emission Tomography and (low dose) Computed Tomography (PET-CT) scan. The study was approved by the Medical Ethical Committee of the Leiden University Medical Center (LUMC) and undertaken in accordance with the principles of the revised Declaration of Helsinki (see the three versions of the study protocol in Data S1. Study protocol v1, related to STAR Methods, Data S2. Study protocol v2, related to STAR Methods, Data S3. Study protocol v3, related to STAR Methods). Written informed consent was obtained from all participants prior to participation. The clinical trial is registered at the Netherlands Trial Register (NTR; NL9345), and at the European Union Drug Regulating Authorities Clinical Trials (EudraCT; 2020-004059-34).

#### Participants

Participants were recruited via emails, flyers and website advertisements. In total, 10 healthy white Caucasian men were enrolled in this study, aged 19 to 35 years and with a body mass index between 19.2 and 26.5 kg/m^2^. Inclusion criteria were: white Caucasian males, age between 18 and 35 years old and BMI ≥18 and ≤25 kg/m^2^. Exclusion criteria were the presence of any endocrine, cardiac, renal, of hepatic disease, a first-degree family member with sudden cardiac death, the use of medication known to influence glucose and/or lipid metabolism, the use of beta-adrenergic receptor agonists (*e*.*g*., for asthma), any contra-indications for the use of salbutamol or propranolol, abuse of alcohol or other substances, smoking, participation in an intensive weight-loss program or vigorous exercise program during the last year before the start of the study, and/or clinically relevant abnormalities in clinical chemistry or electrocardiogram. Eligibility for inclusion was assessed during a screening that consisted of anthropometry, electrocardiography, a questionnaire on medical history, and an overnight 10 h fasted blood sample.

### Method details

#### Randomization

After inclusion, participants (n = 10) were randomized to determine whether they would receive salbutamol in combination with placebo on the first study day (n = 5), or salbutamol in combination with propranolol on the first study day (n = 5). Randomization was executed by the LUMC department of Clinical Pharmacology and Toxicology.

#### Procedures

After inclusion, participants were asked to adhere to several lifestyle rules prior to the study visits: no vigorous exercise 48 h preceding the study days and no alcohol or drinks with caffeine 24 h preceding the study visits. In addition, they were instructed to eat a standardized meal (prepared supermarket meal including pasta or noodles, ranging from 450–600 kcal) in the evening prior to the study visits, and not to eat or drink anything (with an exception for water) afterward until completion of the study visits.

#### Anthropometric measurements

After arrival (9:00 a.m.), body weight (digital balance; E1200, August Sauter GmBH, Albstadt, Germany), height, and waist and hip circumference were obtained. Waist-hip-ratio (WHR) was calculated as: ‘waist circumference’/’hip circumference’. Body composition (fat mass and fat percentage) was estimated using bioelectrical impedance analysis (InBody720, InBody CO., Ltd., CA, USA). In addition, heart rate and blood pressure were measured using a cuff connected to a digital blood pressure device (Model). In total, heart rate and blood pressure were measured at three time points (at the start of the study day, before administration of salbutamol and at the end of the study day). In addition, heart rate was measured at 5 time points (t = 0, 10, 20, 30, 40 min) during the PET scan using a 3-lead ECG connected to a bedside patient monitor (Intellivue MP5, Philips Healthcare, Best, the Netherlands).

#### Indirect calorimetry

At the start and at the end of the study day, resting energy expenditure and substrate utilization were measured for 30 min with a metabolic cart (Vyntus CPX, Carefusion, Hochberg, Germany) equipped with a ventilated hood system that measures total carbon dioxide production (VCO_2_) and oxygen consumption (VO_2_) every 10 s. Before each measurement, volume and gas calibrations were performed. The Weir formula was used to estimate energy expenditure (ignoring urinary nitrogen excretion): energy expenditure (kcal/day) = (3.941∗VO_2_ (L/min)) + (1.106∗VCO_2_ (L/min))∗1440. The first 5 min of gas exchange data of every new recording was discarded, whereafter the most stable 5 min were selected for further analyses, as previously described.^55^

#### Administration of medication

Directly after the first indirect calorimetry measurement, participants received either placebo or 80 mg propranolol *per os*, divided over two capsules each, followed by 75 min of rest to reach peak plasma concentrations of propranolol. The dose, timing and mode of administration are conform the European Association of Nuclear medicine guidelines for tumor imaging.^56^^,^^57^ Afterward, participants were placed in supine position within the PET-CT scanner where salbutamol (250 μg) was administered via a single intravenous bolus (10 mL) into the antecubital vein over a time course of 5 min. The administration of salbutamol intravenously was selected instead of the more commonly used administration via inhalation as high variability in inhalation techniques could not guarantee similar salbutamol exposure in all participants.^58^ In addition, even with an adequate inhalation technique, only a small portion of the administered dose after inhalation will reach the blood.^59^ Currently, the approved dosage of intravenous salbutamol for symptomatic treatment of a severe asthmatic attack is 250 μg.^60^ As salbutamol administered intravenously reaches a maximum concentration within seconds after administration, the medication was injected 15 min prior to the injection of [^18^F] FDG tracer.^58^

#### [^18^F]FDG PET-CT scan

Fifteen minutes after initiation of salbutamol administration, a low dose (30 mA, effective dose 0.7 mSv) CT scan of the cervicothoracic area centered on the supraclavicular region was performed. This was directly followed by the administration of a single bolus of [^18^F]FDG tracer in a dosage of 185 MBq using an injection pump, after which the line was flushed with saline and the dynamic list-mode PET acquisition was started. The list-mode data were reconstructed into 32 time frames (1 × 30 s, 12 × 10 s, 8 × 30 s, 6 × 90 s and 5 × 300 s). The time radioactivity curves for [^18^F]FDG of metabolic tissues were analyzed using the Patlak linearization method,^61^ with the plasma input function taken from the aortic arch.^62^ The slope of the linear phase of the Patlak plot denotes the net influx rate (influx constant, Ki, in min^−1^), which is the accumulated [^18^F]FDG relative to the amount of [^18^F]FDG that has been available in plasma. Ki was then multiplied by circulating glucose levels at the time of the PET image acquisition, and divided by the lumped constant, to calculate the net glucose uptake. Finally, this value was divided by tissue density and multiplied with 1,000 to obtain the net glucose uptake in the preferred unit: nmol·g^−1^·min^−1^. For adipose tissue, the lumped constant of 1.14 and tissue density 0.925 g/mL was used, for skeletal muscle, the lumped constant of 1.16 and tissue density 1.06 g/mL was used.^63^^,^^64^ In summary, the following formula was used to estimate the net glucose uptake by metabolic tissues after intervention:

Netglucoseuptake(nmol/g/min)=((Ki∗circulatingglucose)/(Lumpedconstant))/(Tissuedensity∗1000)

PET-CT image data were analyzed using PMOD software (PMOD technologies LLC, Zürich, Switzerland). Regions of interest (ROIs) were drawn independently by two researchers (M.E.S, C.A.H) on the aortic arch for the plasma input function, four skeletal muscles (*e*.*g*., m. sternocleidomastoid, m. trapezius, m. pectoralis major, m. deltoideus; all left and right), posterior cervical subcutaneous white adipose tissue (scWAT) and supraclavicular BAT (left and right). For skeletal muscles and supraclavicular BAT, values from left and right side were averaged.

#### Blood samples

At the start of the study visit, a catheter was inserted in the antecubital vein, for venous blood sampling and for administration of salbutamol and [^18^F]FDG tracer. At two time points 10 h fasted blood samples were collected: at the start and at the end of the study visit. Blood was collected in Vacutainer SST II Advance tubes. After a clotting time of at least 30 min, samples were centrifuged to obtain serum, which was aliquoted and stored at −80°C until batch-wise analyses. Commercially available enzymatic kits were used to measure serum concentrations of free fatty acids (FFA; Wako chemicals, Nuess, Germany), triglycerides, total cholesterol, high-density lipoprotein cholesterol (HDL-C; all Roche Diagnostics, Woerden, the Netherlands), glucose (Instruchemie, Delfzijl, the Netherlands), insulin and C-peptide (both Meso Scale Diagnostics, Rockville, Maryland, USA). Low-density lipoprotein cholesterol (LDL-C) was estimated using the Friedewald equation.^65^

### Quantification and statistical analysis

#### Sample size

Our power calculation was based on previous studies performed by Blondin et al.^12^ and Orava et al.^54^ Based on these studies, we considered a difference in net glucose uptake rate by BAT of +13 nmol/g/min after salbutamol administration as clinically relevant. From this, we calculated that a sample size of 10 participants would provide a power of 80% to show a clinical relevant effect with a SD of 10.

#### Statistical analysis

Statistical analyses were performed with SPSS Statistics (version 25, IBM Corporation, Armonk, NY, USA). Normal distribution of the data was tested using the Shapiro-Wilk test, visual histograms, and Q-Q plots. To assess the effect of treatment, and to compare the changes after treatment between the treatment regiments, general linear models with repeated measures and pairwise comparisons were used with two within-subject factors: treatment (salbutamol vs. salbutamol with propranolol) and timepoint (*e*.*g*., before and after treatment). Not normally distributed data were log10 transformed (*e*.*g*., energy expenditure, serum FFA, and serum insulin levels). To compare the glucose uptake by BAT and skeletal muscles between treatments, nonparametric Wilcoxon Signed Rank tests were used. To compare the glucose uptake by scWAT between treatments two-tailed paired Student’s t-tests was used. Associations between parameters were tested using Pearson correlations (r) or nonparametric Spearman-rank correlations (rho). Baseline characteristics were compared between non-responders and responders using Mann-Whitney U tests. Absolute changes in heart rate, blood pressure, expenditure, fat oxidation, carbohydrate oxidation, and serum markers were calculated as: ‘end study visit’-‘start study visit’. Changes in energy expenditure were calculated using the following formula: (‘end study visit’-‘start study visit’)/(’start study visit’)∗100%. The homeostasis model assessment-estimated insulin resistance (HOMA-IR) levels were calculated as: ‘fasting glucose in mmol/L’∗‘fasting insulin in μU/mL’/22.5. A p-value of p ≤ 0.05 was considered statistically significant. All data are presented as mean ± SD. Figure 1 was created with BioRender.com. All other figures were prepared with Prism 9 for Windows (version 9.0.1, 2021, GraphPad Software, LLC, San Diego, California, USA).

### Additional resources

Netherlands Trial Register Number (NTR; NL9345) and the European Union Drug Regulating Authorities Clinical Trials Number (EudraCT; 2020-004059-34) (https://www.clinicaltrialsregister.eu/ctr-search/search?query=2020-004059-34).

## Data Availability

• All data reported in this paper will be shared by the lead contact upon reasonable request. • This paper does not report original code. • Any additional information required to reanalyze the data reported in this paper is available from the lead contact upon reasonable request.

## Supplemental information

Document S1. Figures S1–S3 and Table S1

Data S1. Study protocol v1, related to STAR Methods

Data S2. Study protocol v2, related to STAR Methods

Data S3. Study protocol v3, related to STAR Methods

Document S2. Article plus supplemental information
