# Supplementary material for: Stimulation of the beta-2-adrenergic receptor with salbutamol activates human brown adipose tissue
Source: Cell Rep Med. 2023 Feb 21;4(2):100942. doi: 10.1016/j.xcrm.2023.100942 (PMC9975328; doi:10.1016/j.xcrm.2023.100942)
Supplement: Document S1. Figures S1–S3 and Table S1 [file mmc1.pdf]

Cell Reports Medicine, Volume 4

Supplemental information

Stimulation of the beta-2-adrenergic receptor with salbutamol activates human brown adipose tissue

Maaike E. Straat, Carlijn A. Hoekx, Floris H.P. van Velden, Lenka M. Pereira Arias-Bouda, Lauralyne Dumont, Denis P. Blondin, Mariëtte R. Boon, Borja Martinez-Tellez, and Patrick C.N. Rensen

Supplemental data to:

Stimulation of the beta-2-adrenergic receptor with salbutamol activates human brown adipose tissue

Maaike E. Straat[1,2,#], Carlijn A. Hoekx[1,2,#], Floris H.P. van Velden[3], Lenka M. Pereira Arias-Bouda[3], Lauralyne Dumont[4,5], Denis P. Blondin[4,6], Mariëtte R. Boon[1,2], Borja Martinez-Tellez[1,2,Φ], Patrick C.N. Rensen[1,2,Φ,*]

1. Division of Endocrinology, Department of Medicine, Leiden University Medical Center, Leiden, 2333 ZA, the Netherlands.

2. Einthoven Laboratory for Experimental Vascular Medicine, Leiden University Medical Center, Leiden, 2333 ZA, the Netherlands.

3. Section of Nuclear Medicine, Department of Radiology, Leiden University Medical Center, Leiden, 2333 ZA, the Netherlands.

4. Centre de Recherche du Centre Hospitalier Universitaire de Sherbrooke, Sherbrooke, Québec, J1H 5N4, Canada.

5. Department of Physiology-Pharmacology, Université de Sherbrooke, Sherbrooke, Québec, J1H 5N4, Canada.

6. Department of Medicine, Division of Neurology, Université de Sherbrooke, Sherbrooke, Québec, J1H 5N4, Canada

***Corresponding author**: Patrick C.N. Rensen, P.C.N.Rensen@lumc.nl, Leiden University Medical Center, Albinusdreef 2, 2333 ZA, Leiden, The Netherlands

#These authors contributed equally

ΦSenior authors

Clinical Trial Registration: Netherlands Trial Register (NTR), Trial NL9345

Supplementary table 1. Differences in clinical characteristics, serum measurements, and the effect of salbutamol on metabolic parameters between non-responders and responders.

	Non-responders (n=5)	Responders (n=5)
**Glucose uptake by BAT after salbutamol, nmol/g/min**	14.4 ± 3.1	108.7 ± 101.3 **
**Glucose uptake by BAT after salbutamol with propranolol, nmol/g/min**	13.7 ± 2.8	18.4 ± 5.6
**Clinical characteristics**		
**Age, years**	25.6 ± 5.5	23.2 ± 2.7
**Weight, kg**	83.6 ± 8.3	76.1 ± 12.6
**Body mass index**	24.4 ± 1.2	21.9 ± 2.4 #
**Fat mass, %**	16.9 ± 2.3	11.8 ± 1.3 **
**Waist circumference, cm**	84.9 ± 6.3	74.3 ± 7.3 *
**Hip circumference, cm**	96.7 ± 7.5	91.0 ± 8.2
**Waist-hip ratio**	0.9 ± 0.1	0.8 ± 0.02 *
**Systolic blood pressure (start), mmHg**	125.3 ± 10.7	128.9 ± 8.5
**Diastolic blood pressure (start), mmHg**	79.9 ± 7.1	72.4 ± 2.9 #
**Heart rate (start), bpm**	66.5 ± 8.3	77.8 ± 10.0 #
**Heart rate (pre), bpm**	57.8 ± 3.7	62.4 ± 8.2
**Heart rate (end), bpm**	63.6 ± 8.3	72.4 ± 13.5
**Baseline energy expenditure, kcal/h**	2056 ± 68	1887 ± 384
**Serum measurements**		
**Triglycerides, mmol/L**	1.2 ± 0.3	0.8 ± 0.4
**Free fatty acids, mmol/L**	0.7 ± 0.4	0.4 ± 0.2 #
**Total cholesterol, mmol/L**	4.5 ± 0.5	3.0 ± 0.7 **
**HDL-cholesterol, mmol/L**	1.3 ± 0.2	1.0 ± 0.2 #
**LDL-cholesterol, mmol/L**	2.7 ± 0.5	1.6 ± 0.4 *
**Glucose, mmol/L**	5.5 ± 0.1	5.4 ± 0.3
**Insulin, µU/mL**	13.2 ± 5.3	12.3 ± 3.6
**C-peptide, ng/mL**	1.5 ± 0.5	1.6 ± 0.3
**HOMA-IR**	3.2 ± 1.3	2.9 ± 0.8
**Effect salbutamol**		
**Glucose uptake by skeletal muscle, nmol/g/min**	10.5 ± 2.3	8.6 ± 3.5
**Glucose uptake by scWAT, nmol/g/min**	22.2 ± 3.4	20.6 ± 4.3
**Change in energy expenditure, %**	+3.5 ± 3.8	+10.0 ± 12.1
**Change in heart rate, bpm**	+21.6 ± 4.4	+12.2 ± 13.2

Values are presented as mean ± standard deviation. Values are measured at baseline during the salbutamol + placebo-visit. Significance levels are obtained from independent-Samples Mann-Whitney U tests, comparing values from participants that showed a high salbutamol-induced glucose uptake by brown adipose tissue (BAT) (‘responders’) *vs.* participants that showed low salbutamol-induced glucose uptake by BAT (‘non-responders’). #P<0.1, *P<0.05, **P<0.01. HDL, high-density lipoprotein; HOMA-IR, homeostatic model assessment of insulin resistance; LDL, low-density lipoprotein; scWAT, subcutaneous white adipose tissue. Related to **Figure 4**.

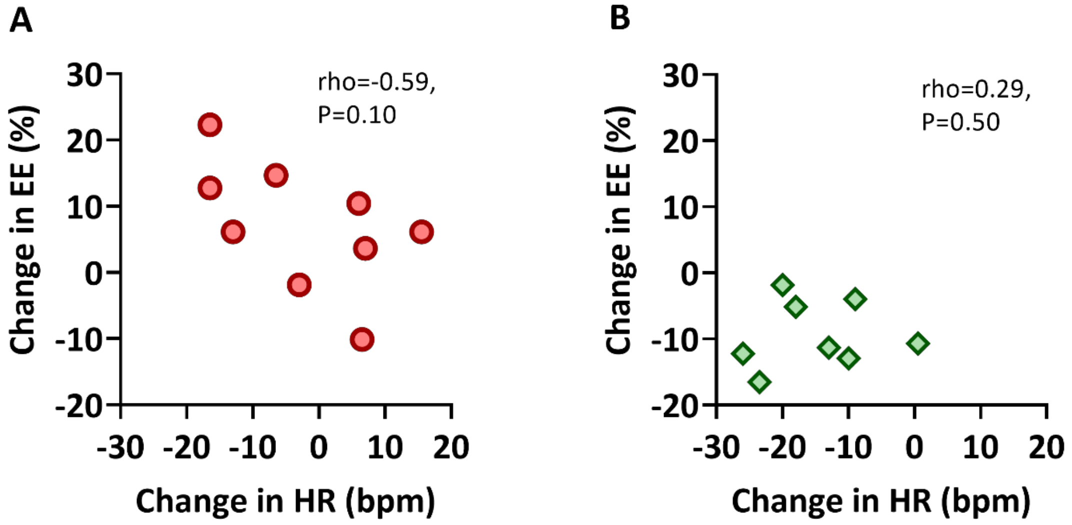

Supplementary figure 1. Correlations plots between the change in energy expenditure (EE) and the change in heart rate (HR) after salbutamol (A) and salbutamol with propranolol (B).

The change in heart rate and EE was calculated as "end study visit" *minus* "start study visit" after salbutamol or salbutamol with propranolol. EE measurement failed due to technical issues for one participant (i.e., missing value, panel A; n=9 red circles). Additionally, the heart rate measurement at the end of the study after salbutamol with propranolol was missing for a second participant (i.e., missing value, panel B; n=8 green diamonds). Related to **Figure 2**.

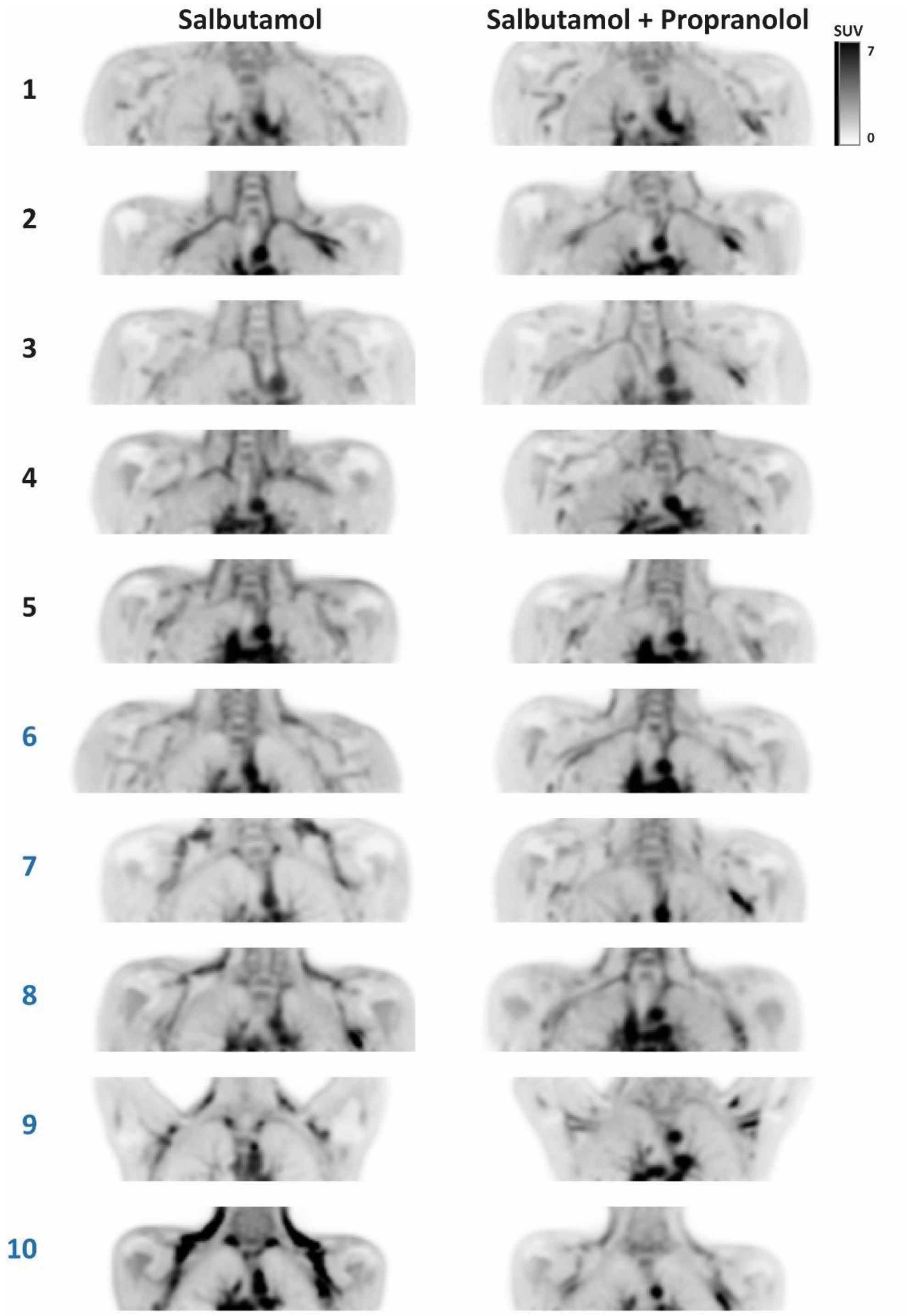

Supplementary figure 2. Tissue 2-[^{18}F]fluoro-2-deoxy-D-glucoseuptake in response to salbutamol and salbutamol with propranolol.

Positron emission tomography (PET) images after salbutamol are shown on the left and PET images after salbutamol with propranolol are shown on the right. Numbers of the scans coincide with the numbers in the waterfall plot of **Figure 4A** and are ordered from lowest (non-responders to salbutamol, black numbers (n=5)) to highest (responders to salbutamol, blue numbers, (n=5)) glucose uptake by brown adipose tissue (BAT). SUV, body-weighted standardized uptake value.

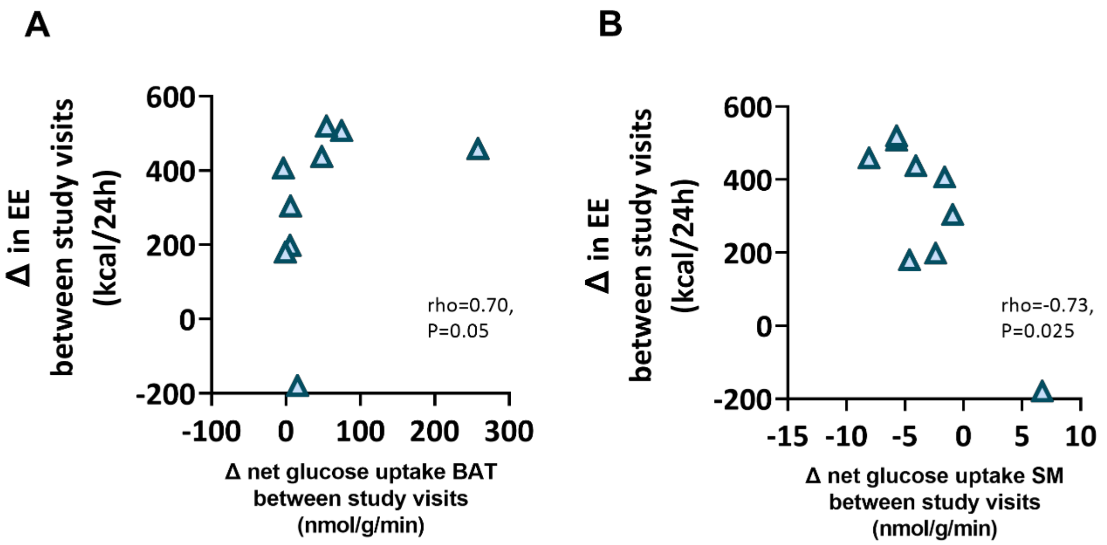

Supplementary figure 3. Correlation plot between the delta in energy expenditure (EE) and the delta in net glucose uptake by brown adipose tissue (BAT; A) or skeletal muscle (SM; B).

All deltas (Δ) were calculated as the change of EE or glucose uptake by brown adipose tissue (BAT) or skeletal muscle (SM) after salbutamol injection *minus* the change of EE or glucose uptake by BAT after salbutamol injection with propranolol. EE measurement failed due to technical issues for one participant (n=9). Related to **Figure 3**.
