# Supplementary material for: Stimulation of the beta-2-adrenergic receptor with salbutamol activates human brown adipose tissue
Source: Cell Rep Med. 2023 Feb 21;4(2):100942. doi: 10.1016/j.xcrm.2023.100942 (PMC9975328; doi:10.1016/j.xcrm.2023.100942)
Supplement: Data S1. Study protocol v1, related to STAR Methods [file mmc2.pdf]

DATA S1: Study protocol v1, related to STAR methods

STIMULATION OF THE BETA-2 ADRENERGIC RECEPTOR FOR ACTIVATING HUMAN BROWN ADIPOSE TISSUE

**Protocol ID**	**NL75061.058.20**
**Short title**	**The beta-2 adrenergic receptor in human BAT**
**Version**	**1**
**Date**	**November 23, 2020**
**Coordinating investigator**	**Drs. M.E. Straat, MD**
**Principal investigator(s)**	**Dr. M.R. Boon**<br>**Dept. of Medicine, Div. of Endocrinology**<br>**M.R.Boon@lumc.nl**
**Co-investigators**	**Prof. Dr. P.C.N. Rensen**<br>**Dr. B.M. Martinez-Tellez**<br>**Dr. L.M. Pereira Arias-Bouda, MD**<br>**Dr. F.H.P. van Velden**<br>**Drs. M.E. Straat, MD**
**Sponsor (in Dutch: verrichter/opdrachtgever)**	**LUMC, Dr. M.R. Boon**
**Independent expert (s)**	**Dr. E.P.M. van der Kleij-Corssmit**
**Laboratory sites**	**Einthoven laboratory, LUMC**<br>**CKCL, LUMC**
**Pharmacy**	**Clinical Pharmacology and Toxicology, LUMC**

PROTOCOL SIGNATURE SHEET

Name	Signature	Date
**Head of Department:**<br>**Prof. dr. A.J. Rabelink**		
**Principal Investigator:**<br>**Dr. M.R. Boon**		

TABLE OF CONTENTS

1. INTRODUCTION AND RATIONALE 10
1.1 Introduction 10
1.2 Brown adipose tissue as target to treat and prevent cardiometabolic disease 10
1.3 Rodents: ADRB3 dominant in activating BAT 10
1.4 Humans: ADRB2 dominant in activating BAT? 11
1.5 Visualization of human BAT 12
1.6 Hypothesis and aims 13
OBJECTIVES 14
2. STUDY DESIGN 15
3. STUDY POPULATION 16
3.1 Population (base) 16
3.2 Inclusion criteria 16
3.3 Exclusion criteria 16
3.4 Sample size calculation 17
4. TREATMENT OF SUBJECTS 18
4.1 Investigational product 18
4.2 Use of co-intervention 18
4.3 Escape medication 18
5. INVESTIGATIONAL PRODUCT 19
5.1 Name and description of investigational product 19
5.2 Summary of findings from preclinical studies 19
5.3 Summary of findings from clinical studies 20
5.4 Summary of known and potential risks and benefits 20
5.5 Description and justification of route of administration and dosage 22
5.6 Dosages, dosage modifications and method of administration 22
5.7 Preparation and labelling of Investigational Medicinal Product 22
5.8 Drug accountability 22
6. NON-INVESTIGATIONAL PRODUCT 24
6.1 Name and description of non-investigational product(s) 24
6.2 Dosages, dosage modifications and method of administration 26
6.3 Preparation and labelling of Non-Investigational Medicinal Product 26
7. METHODS 27
7.1 Study parameters/endpoints 27
7.1.1 Main study parameter/endpoint 27
7.1.2 Other study parameters 27

7.2 Randomization, blinding and treatment allocation 27
7.3 Study procedures 27
7.3.1 Screening 27
7.3.2 Study days 29
7.4 Withdrawal of individual subjects 32
7.5 Replacement of individual subjects after withdrawal 32
7.6 Follow-up of subjects withdrawn from treatment 33
7.7 Premature termination of the study 33
8. SAFETY REPORTING 34
8.1 Temporary halt for reasons of subject safety 34
8.2 AEs, SAEs and SUSARs 34
8.2.1 Adverse events (AEs) 34
8.2.2 Serious adverse events (SAEs) 34
8.2.3 Suspected unexpected serious adverse reactions (SUSARs) 35
8.3 Annual safety report 36
8.4 Follow-up of adverse events 36
8.5 Safety Committee 36
9. STATISTICAL ANALYSIS 37
9.1 Primary study parameter 37
9.2 Other study parameters 37
10. ETHICAL CONSIDERATIONS 39
10.1 Regulation statement 39
10.2 Recruitment and consent 39
10.3 Benefits and risks assessment, group relatedness 39
10.4 Compensation for injury 39
10.5 Incentives (if applicable) 40
11. ADMINISTRATIVE ASPECTS, MONITORING AND PUBLICATION 41
11.1 Handling and storage of data and documents 41
11.2 Monitoring and Quality Assurance 41
11.3 Amendments 42
11.4 Annual progress report 42
11.5 Temporary halt and (prematurely) end of study report 42
11.6 Public disclosure and publication policy 42
12. STRUCTURED RISK ANALYSIS 42
12.1 Potential issues of concern 43
12.2 Synthesis 46
Appendix A. Verantwoording stralingsbelasting 47

13. REFERENCES 49

LIST OF ABBREVIATIONS AND RELEVANT DEFINITIONS

**AE**	**Adverse Event**
**AR**	**Adverse Reaction**
**BAT**	**Brown adipose tissue**
**ADRB2**	**Beta-2-adrenergic receptor**
**ADRB3**	**Beta-3-adrenergic receptor**
**CCMO**	**Central Committee on Research Involving Human Subjects; in Dutch: Centrale Commissie Mensgebonden Onderzoek**
**ECG**	**Electrocardiogram**
**EudraCT**	**European drug regulatory affairs Clinical Trials**
**GCP**	**Good Clinical Practice**
**IC**	**Informed Consent**
**IV**	**Intravenous**
**IMP**	**Investigational Medicinal Product**
**IMPD**	**Investigational Medicinal Product Dossier**
**LUMC**	**Leiden University Medical Centre**
**(S)AE**	**(Serious) Adverse Event**
**SPC**	**Summary of Product Characteristics; in Dutch: officiële productinformatie IB1-tekst**
**SUSAR**	**Suspected Unexpected Serious Adverse Reaction**
**WAT**	**White adipose tissue**
**^{18}F-FDG PET/CT**	**Glucose analogue, 2-[^{18}F]fluoro-2-deoxy-D-glucose positron emission tomography (PET)/computed tomography (CT) scan**

SUMMARY

Rationale:

The prevalence of obesity and associated metabolic diseases is increasing at a disturbing rate. Type 2 diabetes mellitus (T2DM) and cardiovascular diseases (CVD) are currently the leading cause of global death [2]. Obesity is the result of energy intake exceeding energy expenditure. An important contributor to energy metabolism and a promising target to counterbalance the positive energy balance in obesity is brown adipose tissue (BAT) [3]. BAT is a thermogenic organ that is able to combust triglyceride-derived fatty acids and glucose into heat [4, 5]. Naturally, the most well-acknowledged activator of BAT is cold exposure, which provokes an increased sympathetic outflow towards beta-adrenergic receptors on BAT [6, 7]. In rodents, the beta-3-adrenergic receptor (ADRB3) is predominantly found on brown and white adipocytes and activation of the ADRB3 has been shown to effectively activate BAT and improve (cardio)metabolic outcomes [8-10].

Although in humans the ADRB3 agonist mirabegron increases uptake of the positron-emitting glucose analogue 2-[^{18}F]fluoro-2-deoxy-D-glucose ([^{18}F]FDG) by BAT, increases whole body lipolysis and increases resting energy expenditure, this only occurs after administration of a very high dosage of 200 mg [11], which highly exceeds the therapeutic dose to treat hyperactive bladder via ADRB3 activation (i.e. 50 mg). Since at 200 mg also cardiovascular side effects occur, mirabegron probably cross-reacts with ADRB1 and ADRB2 [12]. Furthermore, RNA sequencing analyses on human BAT showed, in contrast to mice, a negligible ADRB3 expression with a high ADRB2 expression. In vitro studies indeed indicate that ADRB2 is responsible activation of human BAT: 1) mirabegron increases oxygen consumption by human brown adipocytes that is inhibited by a specific ADRB2 antagonist, 2) the ADRB2 agonist formoterol activates oxygen consumption by human brown adipocytes, and 3) specific knock-down of ADRB2, and not ADRB1 or ADRB3, reduces oxygen consumption by human brown adipocytes[1]. Whether am ADRB2 agonist is able to activate human BAT *in vivo* has not been investigated yet, but the fact that several studies unequivocally show an increase in resting energy expenditure, lipolysis and lipid oxidation after intravenous (IV) administration of the ADRB2 agonist salbutamol is promising [13-15].

Everything considered, we hypothesize that sympathetic activation of human BAT is mainly mediated by the ADRB2 rather than the ADRB3.

Objectives:

1. To investigate the acute effect of ADRB2 activation, via intravenous administration of salbutamol (250 µg), on [^{18}F]FDG uptake by BAT.
2. To assess the acute effect of ADRB2 activation via intravenous administration of salbutamol (250 µg) on resting energy expenditure, serum markers for lipid- and glucose metabolism and plasma BAT markers.
3. To confirm that the stimulatory effect of salbutamol on [^{18}F]FDG uptake by BAT is not mediated via the ADRB3, by showing that the acute effect of i.v. salbutamol (250 µg) on BAT is blunted by co-administration of the ADRB1/2-blocker propranolol.

Study design:

The study is a randomized double-blinded cross-over trial, that will be carried out at the Leiden University Medical Center (LUMC). This trial encompasses one screening and two study days.

Study population:

This study will be carried out in 10 healthy white Caucasian males between 18 and 35 years old.

Intervention:

For all subjects on both study days the intervention consists of intravenous (IV) injection of salbutamol. This will be combined with either placebo (day 1 or 2) or propranolol (day 1 or 2) oral capsules, which will be given 60 minutes prior to salbutamol injection. In addition, to visualize supraclavicular BAT all subjects will undergo a dynamic [^{18}F]FDG PET/CT scan on both study days.

Main study parameters/endpoints:

- Glucose uptake by BAT, as measured by dynamic [^{18}F]FDG PET/CT acquisition
- Resting energy expenditure, as measured by indirect calorimetry
- Serum markers for lipid metabolism (triglycerides (TG), total cholesterol (TC), high density lipoprotein-cholesterol (HDL-C), low density lipoprotein cholesterol (LDL-C), free fatty acids)
- Lipid pathway analysis using lipidomic analysis in plasma samples
- Serum markers for glucose metabolism (glucose, insulin)
- Circulating plasma BAT markers (*e.g.* microRNAs)

Nature and extent of the burden and risks associated with participation, benefit and group relatedness:

This study consists of three study visits: one screening visit (~1 hour) and two study days (~3.5 hours per day). A total amount of 87.5 mL blood will be drawn, divided over the three visits. In addition, on both study days participants will undergo an [^{18}F]FDG PET/CT scan, with a radiation burden of 8.4 mSv in total. Prior to the PET/CT scan, on both study days

participants will receive 250 µg salbutamol via IV injection, either in combination with placebo or with 80 mg propranolol in capsules. Several pre-cautions regarding the IV administration of salbutamol will be taken into account to prevent from severe side-effects.
Subjects will not directly benefit from participation in this study. However, the results from this study are indispensable for unravelling the working mechanism of human BAT. In addition, the results from this study could reveal new therapeutic targets to activate human BAT and could therefore contribute to the fight against the worldwide obesity epidemic. Therefore, the risks of this study are considered negligible.

1. INTRODUCTION AND RATIONALE

1.1 Introduction

Globally, the prevalence of obesity and associated metabolic diseases is increasing at a disturbing rate, leading to an increased disability and mortality. Since 1975 the worldwide prevalence of obesity has virtually tripled and in 2016 13% of all adults were obese [16]. Importantly, obesity is one of the main risk factors for the development of type 2 diabetes mellitus (T2DM) and cardiovascular diseases (CVD), which are currently the leading causes of global death [2]. Obesity is the result of a disequilibrium between energy intake and energy expenditure. Treatment strategies focusing on decreasing energy intake via diet or increasing energy expenditure via physical exercise are not effective enough in long-term due to incompliance. Therefore, alternative stimulation of mechanisms involved in energy dissipation are a promising target for the treatment of obesity and consequently for the prevention of metabolic diseases.

1.2 Brown adipose tissue as target to treat and prevent cardiometabolic disease

Brown adipose tissue (BAT) has recently come forth as an important factor in energy metabolism. BAT is a highly vascularized thermogenic organ mainly located in the neck region, along the large vessels and in the supraclavicular area. BAT consists of numerous small lipid droplets and a high number of mitochondria. Through the presence of uncoupling protein-1 (UCP-1) in the inner mitochondrial membrane, BAT is able to combust triglyceride-derived fatty acids and glucose into heat and is therewith involved in a process known as thermogenesis [4]. Naturally, the most well-acknowledged activator of BAT is cold exposure. Via cold-sensing receptors in the skin several hypothalamic nuclei in the brain are stimulated, subsequently leading to the release of noradrenalin from sympathetic neurons that binds to adrenergic receptors present on brown adipocytes [6, 7]. Short term cold exposure is known to increase uptake of glucose and fatty acids by BAT, which with an concurrent increase in intracellular release of fatty acids leads to enhanced oxidative energy metabolism in human [17]. Long term cold exposure has been shown to increase energy expenditure and reduce fat mass [18, 19]. Thus, activation of BAT is considered a promising target to counterbalance the positive energy balance provoking obesity and associated cardiometabolic diseases [3]. However, since cold exposure is not a supreme tool for prevention and treatment, alternative targets for activating human BAT are highly demanded. A logical purpose would be to directly stimulate the adrenergic receptors on BAT, to pharmacologically mimic cold exposure.

1.3 Rodents: ADRB3 dominant in activating BAT

In rodents, the beta-3-adrenergic receptor (ADRB3) is predominantly found on brown and white adipocytes[10]. Directly targeting the ADRB3 has been shown to effectively activate

BAT and improve metabolic outcomes [8, 9]. More specifically, activation of the ADRB3 reduces body weight, reduces plasma triglyceride and cholesterol levels, and as a result attenuates atherosclerosis development [8]. Until recently, it was generally assumed that the ADRB3 would also be the most important activating receptor on human BAT.

1.4 Humans: ADRB2 dominant in activating BAT?

Several human studies have in turn aimed to investigate the effect of directly targeting the ADRB3 using mirabegron. The ADRB3 agonist mirabegron is currently approved for the treatment of overactive bladder in a therapeutic dose of 50 mg per day. At the maximal allowable dose, mirabegron (200 mg) indeed increases [^{18}F]FDG uptake by BAT, whole body lipolysis and resting energy expenditure compared to placebo [11]. However, at this dose mirabegron also evokes cardiovascular side effects, such as increases in heart rate (HR) and diastolic and systolic blood pressure (BP), These findings suggest that mirabegron (200 mg) not only stimulated the ADRB3, but also the ADRB1 and possibly ADRB2 that are present on the heart and cardiovascular system [12]. More recent studies investigating the administration of a therapeutic dose (50 mg) of mirabegron show variable results. One study does show an increase of glucose uptake by BAT, albeit to a significantly lower extent compared to the 200 mg dose (18 mL*SUVmean*g/mL versus 306 mL*SUVmean*g/mL, P = 0.007) [20]. Another recent study found no effect of the 50 mg dose on glucose uptake by BAT, nor on BAT oxidative metabolism or BAT blood flow[1].

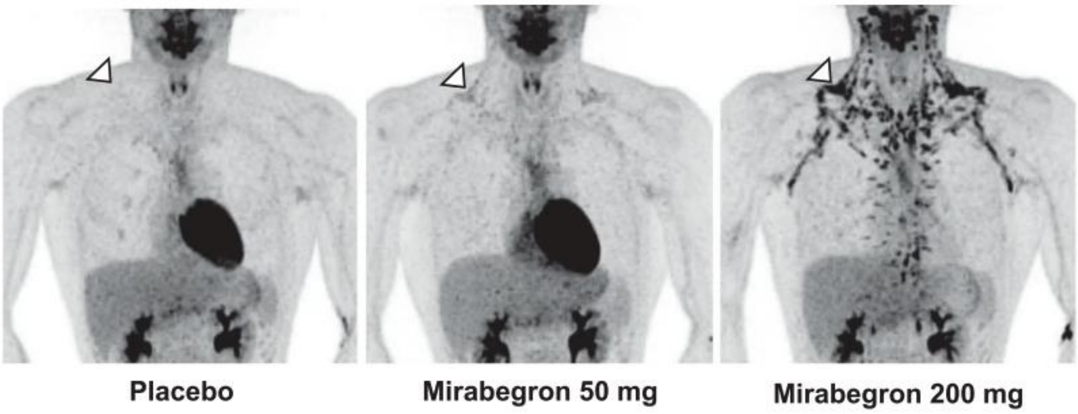

Figure 1 [^{18}F]FDG uptake on PET/CT scan after administration of placebo, 50 mg mirabegron and 200 mg mirabegron.
Baskin et al. Diabetes (2018) [15]

Altogether, this led to the hypothesis that high dose mirabegron activates BAT through cross-reaction with ADRB1 and/or 2, rather than activating ADRB3. Indeed, we recently showed by RNA sequencing a negligible expression of ADRB3 in human BAT, with abundant expression of ADRB2. In vitro studies indeed indicate that ADRB2 is responsible activation of human BAT: 1) mirabegron increases oxygen consumption by

human brown adipocytes that is inhibited by a specific ADRB2 antagonist, 2) the ADRB2 agonist formoterol activates oxygen consumption by human brown adipocytes, and 3) specific knock-down of ADRB2, and not ADRB1 or ADRB3, reduces oxygen consumption by human brown adipocytes[1]. Whether ADRB2 agonism is indeed able to activate human BAT *in vivo* has not been investigated yet, but the fact that several studies do show an increase in resting energy expenditure, lipolysis and lipid oxidation after intravenous (IV) administration of the ADRB2 agonist salbutamol is promising [13-15].

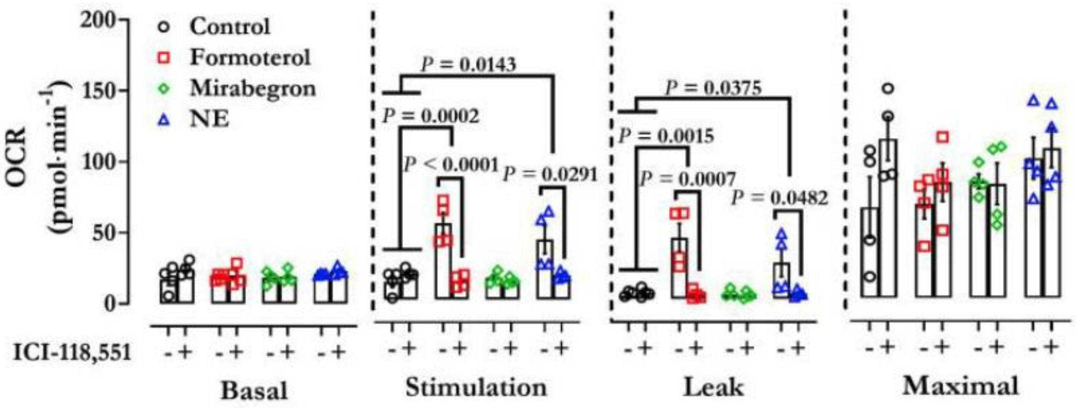

Figure 2 Oxygen consumption rate of in vitro differentiated human brown adipocytes on baseline (Basal), after the addition of formoterol, mirabegron and noradrenaline (Stimulation), leak (Leak) and chemically uncoupled respiration (Maximal). Brown adipocytes were pre-incubated without and with the β2-AR antagonist ICI 118,551. Data are mean ± SEM of n = 4. Blondin et al.[1].

1.5 Visualization of human BAT

The most commonly used method to visualize human BAT *in vivo* is the use of a static positron emission tomography (PET)/computed tomography (CT) scan, with the positron emitting glucose analogue 2-[^{18}F]fluoro-2-deoxy-D-glucose ([^{18}F]FDG) as a tracer. The [^{18}F]FDG tracer is taken up by the sodium-independent glucose transport family (GLUT1, GLUT3 and GLUT4) by metabolically active tissues, amongst which BAT, and can be visualized on PET. The CT scan in turn provides the anatomical reference and distinguishes tissues according to their radiodensity. While many investigators apply static whole-body scans to provide a precise measurement of relative integrated uptake of this tracer between tissues, few have used the dynamic [^{18}F]FDG PET acquisition to precisely determine quantitative measures of BAT glucose uptake rates [21, 22]. Applying a dynamic approach for image acquisition, allows for the derivation of image-acquired, tissue-specific, time-radioactivity curves to determine: fractional tissue-specific glucose extraction (K_i, in min^{-1}), net tissue-specific glucose uptake (K_m, in nmol·g^{-1}·min^{-1}) or if needed the relative integrated uptake of [^{18}F]FDG by the tissue within the period of acquisition (as a static scan would). The fractional glucose extraction and net glucose

uptake rate provide important pharmacokinetic information about the tissue-specific transport of glucose (K_i) and the net uptake of glucose in response to the drug over time. SUV allows for a comparison of the cumulative uptake of glucose between tissues over a fixed period of time. Therefore, dynamic [^{18}F]FDG PET/CT acquisition provides a quantitative and highly sensitive (picomolar range) method to study the effect of a compound on glucose metabolism of human BAT.

1.6 Hypothesis and aims

Everything considered, we hypothesize that sympathetic activation of human BAT is mediated by the ADRB2 rather than the ADRB3.

In this study we aim to investigate the effect of stimulation of the ADRB2 with salbutamol (250 µg) on BAT fractional glucose extraction and net glucose uptake rate by dynamic PET/CT with [^{18}F]FDG. In addition, we aim to show that this effect is abolished when the ADRB1 and 2 are blocked and that this effect is thus not mediated by cross-reaction of salbutamol with the ADRB3. Furthermore, the effect of stimulation of the ADRB2 on resting energy expenditure, plasma lipid and glucose concentrations and plasma BAT markers will be assessed.

OBJECTIVES

Primary Objective:

1. To investigate the acute effect of ADRB2 activation, via intravenous administration of salbutamol (250 µg), on [^{18}F]FDG uptake by BAT.

Secondary Objectives:

2. To assess the acute effect of ADRB2 activation via intravenous administration of salbutamol (250 µg) on resting energy expenditure, serum markers for lipid- and glucose metabolism and plasma BAT markers.
3. To confirm that the stimulatory effect of I.V. salbutamol (250 µg) on [^{18}F]FDG uptake by BAT is not mediated by the ADRB3, by showing that the acute effects of ADRB2 activation by salbutamol is blunted when propranolol (ADRB1/2-blocker) is co-administered.

2. STUDY DESIGN

The study is a randomized double-blinded cross-over trial, that will be carried out at the Leiden University Medical Center (LUMC) (Figure 3). This trial encompasses one screening day, of approximately 1 hour, and two study days, both with a duration of approximately 3.5 hours.

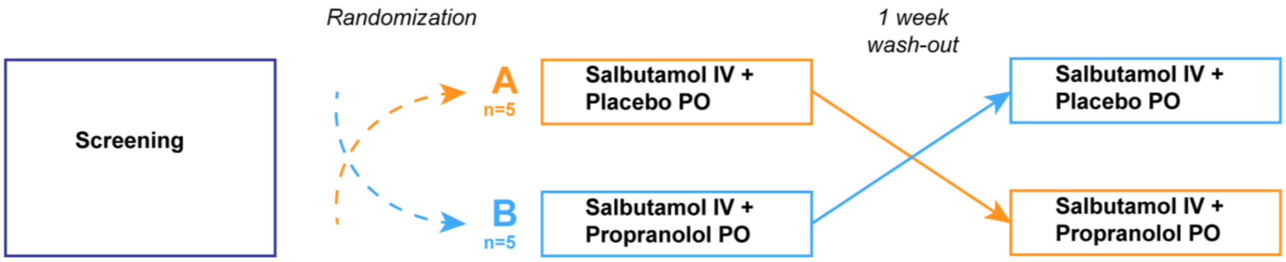

Figure 3 The study is a randomized double-blinded cross-over trial, encompassing one screening day and two study days. IV=intravenous; PO=per oral.

3. STUDY POPULATION

3.1 Population (base)

The study will be carried out in 10 healthy white Caucasian men between 18 and 35 years old. Only young, male subjects will be included to ensure comparability with a currently performed parallel study of our collaborator dr. D.P. Blondin (Université de Sherbrooke, Québec, Canada) who will use the same inclusion criteria. In addition, this will increase comparability with previously performed studies investigating the stimulation of the adrenergic receptors in human BAT activation [11, 20, 23]. Only white Caucasian subjects will be included in this study to enhance homogeneity of the study population, since BAT volume and activity was shown to differ between ethnicities [24]. In case of positive results, the study population should in future studies be expanded with females, other ethnicities or different ranges of BMI, to confirm applicability to the general population.
All subjects will be recruited via advertising at the LUMC and in university buildings in Leiden. In addition social media, such as Facebook and LinkedIn, and the website of the Dutch Heart Foundation will be used to advertise.

3.2 Inclusion criteria

To be eligible to participate in this study, a subject must meet all the following criteria:

- Dutch white Caucasian males
- Age between 18-35 years old
- Lean (BMI ≥ 18 and ≤ 25 kg/m^2)

3.3 Exclusion criteria

A potential subject who meets any of the following criteria will be excluded from participation in this study:

- Diabetes mellitus (determined on basis of fasting glucose levels defined by ADA criteria)
- Any other active endocrine disease (thyroid disease, any signs of Cushing’s syndrome, adrenal disease and lipid-associated disorders such as familial hypercholesterolemia)
- Any cardiac disease (i.e. ischemic cardiac disease, arrhythmias, severe heart failure)
- A first-degree family member with sudden cardiac death
- Any chronic renal or hepatic disease
- Use of beta-adrenergic receptor agonists (for *e.g.* asthma)
- Use of medication known to influence glucose and/or lipid metabolism or brown fat activity (e.g. beta-blockers, antidepressants, corticosteroids)
- Use of medication shown to increase risk on hypokalemia after salbutamol administration (e.g. xanthine derivatives, steroids and diuretics)

- Any other contra-indications for the use of salbutamol or propranolol
- Abuse of alcohol or other substances
- Smoking
- Participation in an intensive weight-loss program or vigorous exercise program during the last year before the start of the study
- Current participation in another research projects that may influence the current research project
- Participation in another research with exposure to radiation burden within a year before the start of the current study
- Clinically relevant abnormalities in clinical chemistry or electrocardiogram (ECG) at screening (to be judged by the study physician)

3.4 Sample size calculation

For this study the main outcome parameter is net glucose uptake by BAT after administration of salbutamol, as measured by dynamic ^{18}F-FDG PET-CT scan.

Our sample size calculation is based on the results recently published by Blondin et al.[1] and Orava et al. [25]. Blondin et al. showed net glucose uptake by BAT as measured by dynamic ^{18}F-FDG PET/CT after administration of the maximal allowable dose mirabegron (200 mg) of 22±14 nmol/g/min. We expect the effect of salbutamol on glucose uptake by BAT to be at least as big as the effect of the maximal allowable dose mirabegron. Orava et al. [25] showed net glucose uptake by BAT in thermoneutral condition of 9±4 nmol/g/min. Therefore, we predict the net glucose uptake of salbutamol in combination with propranolol to be comparable to the net glucose uptake by BAT in thermoneutral condition. Thus, we consider a difference in net glucose uptake by BAT of +13 nmol/g/min after salbutamol administration as clinically relevant, with a SD of 10, $\alpha = 0.01$, $\beta = 80\%$. Based on these parameters we need 10 subjects (http://biomath.info/power/prt.htm; based on Snedecor & Cochran, Statistical methods 7th edition).

In case a subject is withdrawn from the study before finishing all measurements, the investigator will try finding a replacement to maintain the initial population size.

4. TREATMENT OF SUBJECTS

This is a randomized double-blind cross-over trial in young healthy men. For all subjects the intervention consists of IV injection of salbutamol in combination with placebo and, as comparator, IV injection of salbutamol in combination with propranolol, in random order. In addition, at both study days all subjects will undergo a low-dose dynamic ^{18}F-FDG PET/CT scan.

4.1 Investigational product

The investigational product in this study is salbutamol. During the two study days subjects will receive a single-dose of 250 µg salbutamol via IV injection over a time-course of 5 minutes. Salbutamol is a selective ADRB2 agonist approved for the symptomatic treatment of asthma and chronic obstructive pulmonary disease (COPD) [26]. More information on salbutamol will be included in the following section 'Investigational product'.

Administration of salbutamol will be given in combination with placebo (study day A) or in combination with 80 mg propranolol (study day B) (Figure 3 and 4). Placebo and propranolol will be given orally in two capsules 60 minutes prior to salbutamol injection and are solely used to further elucidate which receptors are involved in the working mechanism of salbutamol on human BAT. Therefore, placebo and propranolol are not considered investigational products. More information about placebo and propranolol will be provided in section 7 'Non-investigational product'.

4.2 Use of co-intervention

Not applicable

4.3 Escape medication

Not applicable

5. INVESTIGATIONAL PRODUCT

5.1 Name and description of investigational product

In this study subjects will receive a single dose of 250 µg salbutamol by IV injection over the time-course of 5 minutes on both study days to investigate the effect of salbutamol on glucose uptake by human BAT. Salbutamol is a selective ADRB2 agonist used for the prevention and symptomatic treatment of bronchospasms in asthma and chronic obstructive pulmonary disease (COPD). Administration of salbutamol in IV form is approved in clinic for the acute treatment of severe asthmatic attacks and status asthmaticus[27]. Our study protocol is based on a study recently performed by Kruizinga et al. [28] in the Centre for Human Drug Research (CHDR) in Leiden. In this study, IV salbutamol in a dosage of 500 µg was proven to be safe and well-tolerated. Salbutamol will be provided by the LUMC department Clinical Pharmacy and Toxicology.

5.2 Summary of findings from preclinical studies

The rationale of this study is based on extended preclinical and clinical research showing that BAT is most potently activated through the sympathetic nervous system, which acts via adrenergic receptors present in adipose tissue. This research shows that in rodents ADRB3 mRNA is predominantly found in high levels in brown adipocytes [10]. Directly targeting the ADRB3 using CL316243, one the most selective ADRB3 agonists available, was shown to effectively activate BAT and improve cardiometabolic outcomes [8, 9]. More specifically, activation of the ADRB3 reduces plasma triglyceride and cholesterol levels, decreases body weight and consequently attenuates atherosclerosis development [8]. The potential benefit of activating BAT via stimulation of adrenergic receptors is thus clearly illustrated in preclinical research. However, in human studies directly targeting the ADRB3 has thus far led to inconsistent results [11, 12, 20]. In addition, a recent study shows that human BAT shows negligible expression of ADRB3 with abundant expression of ADRB2[1]. Therefore, in this study we aim to investigate the effect of selectively targeting the ADRB2 using salbutamol. Preclinical studies investigating the effect of salbutamol on brown adipose tissue are scarce, due to the low expression of the ADRB2 in rodent BAT. One study showed no direct effect of salbutamol on brown adipocytes, but an increased glucose uptake by BAT in mice due to increased blood flow through the tissue [29]. In addition, a recent study investigated exclusively peripheral stimulation of the ADRB2, using PEGyAMPH. They showed increased sympathetic outflow towards WAT and BAT, leading to increased lipolysis and thermogenesis [30]. Altogether, it is very plausible that, while in mice the ADRB3 is the most important adrenergic receptor for the activation of BAT, in humans the ADRB2 in fact activates BAT. Other non-clinical studies investigating the selective stimulation of the ADRB2 with salbutamol or other

compounds mainly focused on pulmonary and cardiovascular outcomes and will therefore not be summarized here. For findings from preclinical studies regarding pharmacological safety of salbutamol we refer to the 'Summary of Product Characteristics' (SPC), which can be found on the website of the 'College ter Beoordeling van Geneesmiddelen' (CBG) (https://www.geneesmiddeleninformatiebank.nl/nl/, registration number RVG 07686) and to the product specification in appendix D2.

5.3 Summary of findings from clinical studies

Salbutamol is approved in clinical care since 1968 for the prevention and treatment of bronchospasms in asthma and chronic obstructive pulmonary disease (COPD) and is thus extensively studied regarding cardiopulmonary outcomes. A few studies have focused on the effect of salbutamol on energy metabolism [13-15, 31]. However, none of them specifically focused on human BAT.

The ADRB2 is one of the three subtypes of beta-adrenergic receptors, which are well-acknowledged to be important for the regulation of energy metabolism [32]. Several studies showed that selective stimulation of the ADRB2 using IV administration of salbutamol, with or without blocking the ADRB1 using atenolol, increases energy expenditure, lipid oxidation and lipolysis [13-15, 31]. This effect appears to be lower in obese compared to lean subjects [13] and is possibly affected by different polymorphisms of the ADRB2 [15]. These results are in line with our hypothesis that specifically stimulation of the ADRB2 will lead to increased activation of human BAT, which in turn leads to increased energy expenditure, lipid oxidation and lipolysis.

5.4 Summary of known and potential risks and benefits

Findings from clinical studies regarding pharmacological safety and potential adverse effects of salbutamol can be found in the 'Summary of Product Characteristics' (SPC), which can be found on the website of the 'College ter Beoordeling van Geneesmiddelen' (CBG) (https://www.geneesmiddeleninformatiebank.nl/nl/, registration number RVG 07686) and in the product specification in appendix D2. In addition, a structured risk analysis can be found in paragraph 13 'Structured risk analyses'. We will here discuss the main side effects of concern and the precautions we will be undertaking. At all times there will be a medical doctor and nurse present during the study days. The study protocol regarding the route of administration of salbutamol and the to be undertaken precautions is based on a study recently performed by Kruizinga et al. [28] at the Centre for Human Drug Research (CHDR) in Leiden.

Cardiovascular side effects

Sympathomimetic drugs such as salbutamol can induce cardiovascular effects, often

(>10%) including tachycardia and palpitations and rarely (0.01-0.1%) including arrhythmias [26]. This could potentially be a risk for patients suffering from ischemic cardiac disease, arrhythmias or severe heart failure. Therefore, we screen all subjects on cardiac abnormalities using an ECG during the first screening visit. In addition, family history regarding cardiac diseases or sudden death will be interrogated. In case of any cardiac abnormalities subjects will be excluded for participation in this study. In addition, during the study day we will monitor heart rate and blood pressure using a cuff connected to a digital blood pressure device at three set time points (at the start of the study day, before administration of salbutamol and after PET/CT scan), and we will monitor heart rate and rhythm during PET/CT scan using a 3-lead ECG connected to a bedside patient monitor. Before administration of salbutamol we accept a heart rate < 100 beats per minute and a systolic blood pressure <140 mmHg. Previously performed studies in healthy subjects report a mean increase of ca. 15 heart beats per minute after continuous IV salbutamol administration (50-100 ng/kg fat free mass per minute) [13, 15]. In this study we will administer salbutamol intravenously via a single bolus. We expect the initial increase in heart rate to be comparable to the rates mentioned in the aforementioned studies and we expect this to decline over-time to baseline levels after administration [33].

Hypokalemia

Hypokalemia may occur after IV administration of salbutamol, an effect that is potentiated by hypoxia and concomitant treatment with xanthine derivatives, steroids and diuretics [26]. In this study we will only include healthy subjects that are not suffering from bronchospasms and are thus not hypoxic. In addition, we will exclude subjects that use one of the aforementioned drugs. During the screening visit, at the start of the study day and at the end of the study day we will monitor serum potassium levels.

Lactic acidosis

Lactic acidosis has very rarely (<0.01%) been reported after administration of high doses of IV salbutamol [26]. High levels of lactate could lead to dyspnea and compensatory hyperventilation and Kussmaul breathing. Therefore, study physicians will be aware of any signs of dyspnea (e.g. tachypnoea). However, the occurrence of lactic acidosis seems unlikely in our study design in which we only inject a single dose of salbutamol, since acidosis is shown to quickly resolve after reduction or termination of salbutamol administration [34].

5.5 Description and justification of route of administration and dosage

In this study we will administer 250 µg salbutamol via an IV single dose injection over 5 minutes.

Salbutamol is available and approved for clinical use in inhalation, intramuscular, subcutaneous and IV form. Salbutamol inhalation is most commonly used in clinic and particularly works locally in the bronchopulmonary tract. However, in this study we are interested in the effect of salbutamol on BAT activation. Only a small proportion of the administered dose after inhalation will enter the blood stream [28, 35], and is thus able to cause an effect in adipose tissue. In addition, the variability in inhalation techniques causes variable plasma concentrations of the drug after inhalation [28]. This high variability in plasma concentrations between subjects could lead to an inconsistency in pharmacological activity at the effect site. Salbutamol by IV injection or infusion is approved for the symptomatic treatment of severe asthmatic attacks or status asthmaticus, in a minimum dose of 250 µg or 5 µg/min IV respectively [26]. Previously performed studies investigating the effect of salbutamol on energy metabolism have all used IV administration of salbutamol [13-15, 28, 31]. After IV administration plasma concentrations appear to be rather consistent [28]. Kruizinga et al. [28] used a single dose injection of 250 µg and 500 µg salbutamol in their study protocol and did not experience severe side effects. Almost all subjects became moderate tachycardic which lasted for around 30 minutes, however, subjects were informed about this beforehand and none of them experienced this as highly uncomfortable. In the previous section (6.4 Summary of known and potential risks and benefits) we summarized the potential risks and the precautions we will be undertaking to limit potential risks to a minimum.

5.6 Dosages, dosage modifications and method of administration

Salbutamol will be administered intravenously in a single dose injection of 250 µg over the time course of 5 min.

5.7 Preparation and labelling of Investigational Medicinal Product

Preparation and labelling of salbutamol will be done by the department Clinical Pharmacy and Toxicology of the LUMC according annex 13 of the guideline Good Manufacturing Practice (2003/94/EG, via http://ec.europa.eu/health/files/eudralex/vol-4/2009_06_annex13.pdf) and local law and trial requirements. An example of the label is provided in section D ‘Product information’.

5.8 Drug accountability

The principal investigator will be responsible for drug accountability and will ensure that the whole research team understands and follows the research protocol. Drug accountability will be cared for by the department Clinical Pharmacy and Toxicology of

the LUMC. The drug accountability record will be filled in during the study days by the sub-investigator and research nurse running the clinical trial. The trial product will only be dispensed to subjects in the trial according to the protocol.

6. NON-INVESTIGATIONAL PRODUCT

6.1 Name and description of non-investigational product(s)

In this study we use non-investigational products: propranolol, placebo, the radioactive [^{18}F]FDG tracer and several medical devices.

Propranolol and placebo

During the two study days administration of our investigational product salbutamol will be preceded with either placebo (study day A) or 80 mg propranolol (study day B), given orally divided over two capsules (Figure 3 and 4). Propranolol is a non-selective ADRB1/2 blocker, approved and commonly used in clinical care for the treatment of hypertension, secondary prophylaxis after a myocardial infarction, angina pectoris, hypertrophic obstructive cardiomyopathy, arrhythmias, thyrotoxicosis and as prophylaxis for migraine [36]. In literature the use of propranolol is regarded to be effective to suppress BAT in patients undergoing an [^{18}F]FDG PET/CT for oncology reasons [37-40]. Propranolol administered in a dosage of 80 mg orally two hours before [^{18}F]FDG PET/CT acquisition was shown to almost completely suppress BAT activity [40]. This same method is also mentioned in the guidelines for tumor imaging from the European Association of Nuclear Medicine [41].

In this study, propranolol will be used to reverse activation of BAT after administration of salbutamol by antagonizing its effect on the ADRB1 and B2. The ultimate goal is to show that the effect of salbutamol on BAT is hereupon abolished and thus not mediated via overspill towards the ADRB3.

For a summary of findings from non-clinical and clinical studies regarding pharmacological safety of propranolol we refer to the Summary of Product Characteristics (SPC), which can be found on the website of the ‘College ter Beoordeling van Geneesmiddelen’ (CBG) (https://www.geneesmiddeleninformatiebank.nl/nl/, registration numbers RVG 12638) and to the product specification in appendix D2.

^{18}F-fluorodeoxyglucose (^{18}F-FDG) tracer

After administration of placebo or propranolol together with salbutamol a dynamic PET/CT scan will be performed with the use of the radioactive tracer [^{18}F]FDG in a dosage of 185 MBq. [^{18}F]FDG is a positron-emitting glucose analogue that is taken up by the sodium-independent glucose transport family (GLUT1, GLUT3 and GLUT4) in the cell membrane of metabolically active tissues. Due to a radioactive label these metabolically active tissues can in turn be visualized using a PET scan. The CT scan in addition provides the anatomical information. The [^{18}F]FDG PET/CT scan is a non-invasive

diagnostic device that is used in clinical care for the quantification of the metabolic activity of tissues. Indications for [^{18}F]FDG PET/CT include, but are not limited to, the detection, differentiation and classification of oncologic malignancies and the detection of inflammatory processes.
The [^{18}F]FDG tracer will be delivered by GE Healthcare B.V. (Steripet®, Eindhoven, the Netherlands). For more information about the [^{18}F]FDG tracer we refer to the summary of product characteristics (SPC), which can be found on the website of the 'College ter Beoordeling van Geneesmiddelen' (CBG) (https://www.geneesmiddeleninformatiebank.nl/nl/, registration numbers RVG 29834) and to the product specification D2.

Medical devices
Table 1 provides an overview of the devices considered as non-investigational medical products (non-IMPs) that are included in this study: PET/CT, the body composition analyzer, the infrared camera and indirect calorimeter. The device, name, manufacturer and LUMC inventory numbers are provided for each non-IMP. All devices are CE marked and will be utilized within their intended use. Further information is provided in the intake forms for clinical research with medical devices.

Table 1 Overview of non-IMP devices

Device	Name	Manufacturer	LUMC numbers
**ECG (Screening)**	ELI™ 280 Resting Electrocardiograph	WelchAllyn, Mortara	16-800-1692
**Body composition analyzer**	InBody720	InBody CO., Ltd.	12-091-0149
**Bedside monitor including ECG (Study days)**	Philips Intellivue MP5	Philips Healthcare, Best, The Netherlands	17-800-1688
**Indirect calorimeter (2x)**	Vmax Vyntus CPX	CareFusion	17-800-0138 & 17-800-0137
**PET/CT scanner**	Vereos version 2.0.2.26321	Philips Healthcare, Best, The Netherlands	17-840-0074

6.2 Dosages, dosage modifications and method of administration

Propranolol and placebo

Propranolol or placebo will be administered orally 1 hour before the start of the [^{18}F]FDG PET/CT scan. Dosage of propranolol will be 80 mg, divided over two capsules of 40 mg. Placebo will consist of two similar looking capsules as well.

[^{18}F]FDG

A bolus of [^{18}F]FDG tracer will be intravenously injected in a dosage of 185 MBq directly prior to the start of the PET/CT scan.

6.3 Preparation and labelling of Non-Investigational Medicinal Product

Propranolol and placebo

Propranolol and placebo will be blindly packed and labelled by the LUMC department Clinical Pharmacy and Toxicology, according annex 13 of the guideline Good Manufacturing Practice (2003/94/EG, via http://ec.europa.eu/health/files/eudralex/vol-4/2009_06_annex13.pdf) and local law and trial requirements. An example of the label is provided in section D ‘Product information’.

[^{18}F]FDG

[^{18}F]FDG is obtained and delivered by GE Healthcare B.V. (Steripet®, Eindhoven, the Netherlands) at the LUMC. In total, for every subject 185 MBq [^{18}F]FDG is needed. FDG will be ordered by the Nuclear Medicine section of the Radiology department of the LUMC, on request of the research physicians. [^{18}F]FDG will be delivered calibrated at an indicated time point for an indicated subject by GE Healthcare B.V. (Steripet®, Eindhoven, the Netherlands) in standard packaging to the department of Radiology, section of Nuclear Medicine, at LUMC.

7. METHODS

7.1 Study parameters/endpoints

7.1.1 Main study parameter/endpoint

Glucose uptake by BAT, as measured by dynamic [^{18}F]FDG PET/CT acquisition

7.1.2 Other study parameters

- Resting energy expenditure, as measured by indirect calorimetry
- Serum markers for lipid metabolism (triglycerides (TG), total cholesterol (TC), high density lipoprotein cholesterol (HDL-C), low density lipoprotein cholesterol (LDL-C), free fatty acids)
- Lipid pathway analysis using lipidomic analysis in plasma samples
- Serum markers for glucose metabolism (glucose, insulin)
- Circulating plasma BAT markers (*e.g.* microRNAs)

7.2 Randomization, blinding and treatment allocation

Subjects will be randomized to decide whether they will receive salbutamol in combination with placebo on the first study day (group A), or salbutamol with the addition of propranolol (group B) (see also Figure 3, in chapter 3. 'Study design'). Randomization will be executed by the LUMC department of Clinical Pharmacology and Toxicology. In this study there will be two randomization groups (A and B) with the weight 1:1 divided over two blocks with a size of 5. We will not use stratification. The study will be performed double-blindly.

7.3 Study procedures

This study encompasses three visits: one screening visit and two study days.
First subjects will participate in the screening procedure. The screening consists of the informed consent procedure, a medical history questionnaire, anthropometric measurements, a blood draw and an ECG.

7.3.1 Screening

Inclusion and informed consent procedure

Subjects will be recruited via flyers (see E3), both in the LUMC and in university buildings in Leiden. Hereupon they can approach the researchers via email or telephone. Their eligibility is assessed with a short telephonic interview checking the basic inclusion criteria, after which patient information will be sent by regular mail or email including the informed consent form (see document E1/E2) and flyer

'Medisch-wetenschappelijk onderzoek. Algemene informatie voor de proefpersoon'. After one week of consideration about their decision a screening appointment will be planned. The screening appointment starts with discussing the study information and informed consent form once again in person with the researcher. Thereafter the informed consent form will be signed by the participant and the researcher.

Screening visit

The rest of the screening visit will include:

- Demographics and medical history
- Anthropometric measurements
- Fasting blood sample
- ECG

An overview of all elements of the screening visit is given in table 2. The subject will be included in the study in case he meets all the inclusion criteria, is willing to participate in the study, and has signed the informed consent.

Table 2 Overview of the screening visit

	**Items**
**Demographics and medical history**	Age<br>Comorbidities<br>Current and previous used medication<br>Family history
**Anthropometric measurements**	Body weight<br>Body height<br>Blood pressure, heart rate<br>Body composition (using the Body composition analyzer InBody720)
**Fasting blood sample**	Venapuncture for determination of fasting blood glucose, insulin, TC, TG and parameters of liver (ASAT, ALAT, gGT), kidney (creatinine), thyroid function (TSH), electrolytes (including potassium) and a full blood count.<br><br>A total of 7.5 mL blood is withdrawn in a SST®II Advance Gel tube of 3.5 mL and an EDTA containing tube of 4 mL, all from Becton and Dickinson®. These samples will be analyzed at the Department of Clinical Chemistry in the LUMC.
**ECG**	12-lead ECG

7.3.2 Study days

This study encompasses two study days, both will take place in the LUMC. Subjects will be randomized to decide whether they will receive salbutamol in combination with placebo first (study day A) or salbutamol in combination with propranolol (study day B). All measurements during the two study days are identical. Both study days will be performed in the morning after an overnight fast. This means that subjects are not allowed to drink (except water) and eat from 10 PM the evening before, until arrival in the morning. Furthermore, subjects will be asked:

- not to exercise 48 hours in advance;
- not to drink alcohol 24 hours in advance;
- not to drink tea or coffee 24 hours in advance;
- to eat a standardized meal the evening before (options will be given by the researcher).

An overview of the timeline of the two study days and the involved study procedures is given in figure 4.

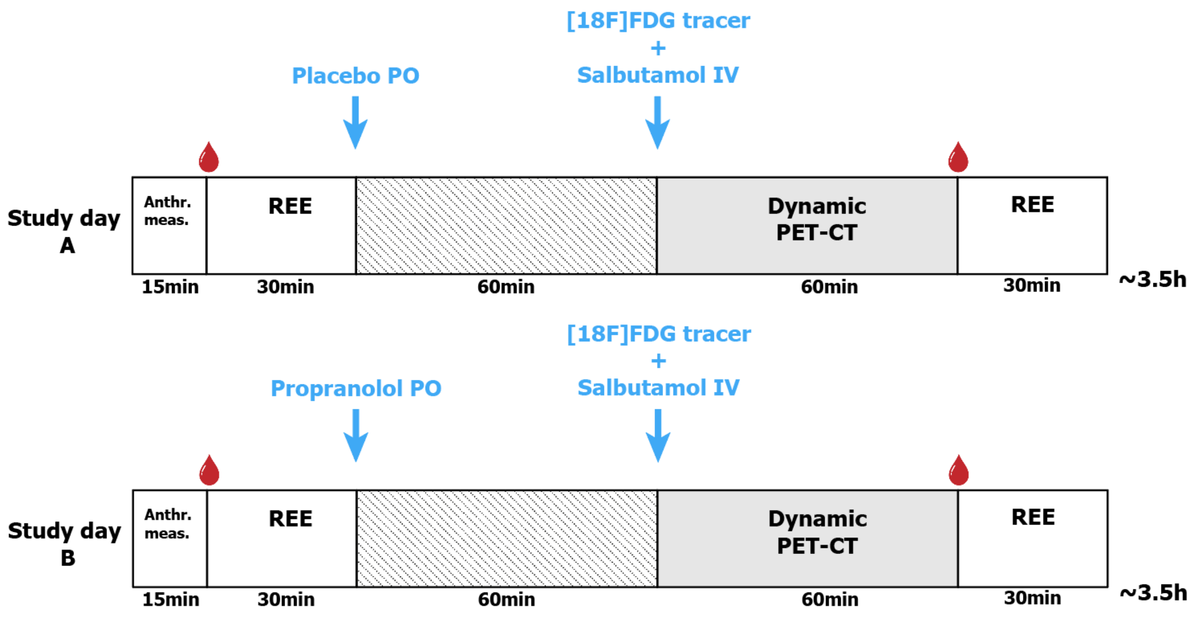

Figure 4 Study procedures.
After arrival anthropometric measurements (including heart rate and blood pressure) will be determined. An intravenous catheter will be placed and a baseline blood sample will be drawn. This will be followed by a resting energy expenditure (REE) measurement for 30 minutes using indirect calorimetry. Directly after the REE measurement subjects will receive either placebo (study day A) or propranolol (study day B). After 60 minutes, heart rate and blood pressure will be measured again (not shown in figure). Then subjects will be placed in the PET/CT scanner and salbutamol will be administered, directly followed by [^{18}F]FDG insertion. First, a low dose CT scan will be performed, followed by dynamic PET acquisition for 60 minutes. While scanning heart rate and rhythm will continuously be monitored. After PET/CT acquisition a blood sample will be drawn, heart rate and blood pressure will be measured and finally REE will be measured for another 30 minutes.

Anthropometric measurements

Shortly after arrival (8:00 AM), anthropometric measurements will be performed (weight, height, waist and hip circumference) and heart rate and blood pressure will be measured.

Blood sampling

Next, in both arms a catheter will be inserted in an antecubital vein, for venous blood sampling and for administration of salbutamol and [^{18}F]FDG tracer later during the study day. At two timepoints fasted blood samples will be collected: at the start of the study day and after PET/CT acquisition. In these samples we will determine markers for lipid metabolism (TC, FFA, TG, HDL-C, LDL-C, lipidomics) and glucose metabolism (glucose, insulin). Blood for these measurements will be collected in SST®II Advance Gel tubes of 8 mL and blood collection tubes containing ethylenediaminetetraacetic acid (EDTA) of 8 mL. In addition, we will measure electrolytes (sodium, potassium) in a heparin-lithium tube of 4 mL.

The heparin-lithium tube will directly be brought to the Department of Clinical Chemistry in the LUMC to quickly obtain information about electrolyte levels. The EDTA tube will after blood collection immediately be put on ice and centrifuged promptly (2,000 g at 4°C, for 10 minutes). The SST®II Advance Gel tubes must coagulate for 30-45 minutes before centrifugation. After centrifugation plasma and serum from both tubes will be divided in separate plastic tubes and frozen (-80°C) until assay. Material of all included patients from the SST®II Advance Gel tubes and EDTA tubes will be collected first and then above research markers will be determined all at once at the Department of Endocrinology of the LUMC. The ‘rest material’ will be stored in the LUMC Biobank Endocriene Ziekten for later additional analysis. For this material the rules and regulations of the LUMC Biobank Endocriene Ziekten are applicable.

An overview of the blood collection during the screening and the study days is provided in table 3.

Table 3 Blood collection during screening visit and study days

Timepoint	Measurement	Tubes	Amount
**Screening**	TC, TG, FFA, Lipoproteins, Glucose, Insulin, Liver-, kidney-, Thyroid function, Electrolytes, Full blood count	SST®II Advance Gel tube (3.5 mL)<br>EDTA tube (4 mL)	7.5 mL
**Study days**	TC, TG, FFA, Lipoproteins, Glucose, Insulin, Sodium, Potassium, Lipidomics,<br>BAT markers (miRNAs)	SST®II Advance Gel tube (8 mL)<br>EDTA tube (8 mL)<br>Heparin-lithium tube (4 mL)	20 mL per timepoint<br><br>4x20 mL = 80 mL
			**87.5 mL**

Energy expenditure measurement

At two time points (after the first blood sample and after [^{18}F]FDG PET/CT scan) resting energy expenditure will be measured for 30 minutes with a respiratory gas analyzer using a ventilated hood system (Vyntus Canopy). The volume of inspired oxygen and exhaled carbon dioxide will be measured every minute. Using previously described formulas resting energy expenditure will be calculated [42].

Administration of medication

When the first resting energy expenditure measurement has finished, subjects will receive either placebo or 80 mg propranolol per oral. Propranolol will be completely absorbed after oral administration and reaches peak plasma concentrations after 1-2 hours. Therefore, administration of placebo or propranolol will be followed by an hour of rest. Subjects can bring something for entertainment (i.e. a book, magazine, music, etc.), but will not be left alone without supervision of a sub-investigator or research nurse.

After an hour of rest, the study day will continue. Vital signs will be measured again to check whether heartrate and systolic blood pressure meet the accepted values of <100 beats per minute and <140 mmHg, respectively. Then subjects are placed in supine position within the PET/CT scanner and salbutamol will be administered via an

IV injection over a time course of 5 minutes. Administration of salbutamol is directly followed by the administration of a bolus of [^{18}F]FDG tracer in a dosage of 185 MBq using an injection pump.

Dynamic [^{18}F]FDG *PET/CT*

A dynamic [^{18}F]FDG PET/CT scan will be performed to determine glucose uptake by supraclavicular BAT. Scans will be performed on a digital PET-CT scanner (Vereos; Philips Healthcare, Best, The Netherlands) available at the department of Radiology, section of nuclear medicine, of the LUMC in collaboration with Nuclear medicine physician Dr. L.M. Pereira Arias-Bouda and medical physicist dr. F.H.P. van Velden. The scanning protocol is based on the protocol regularly used by our collaborator Dr. D.P. Blondin[17]. First a low dose (30 mA, effective dose 0.7 mSv) CT scan of cervicothoracic area centered on the supraclavicular region will be performed, to locate PET regions of interest and used for attenuation correction of the PET-image. This will immediately be followed by a 60 min dynamic list-mode PET acquisition. For two scans this will lead to a total radiation burden of 8.4 mSv, which is below the threshold of 10 mSv [appendix A]. The list-mode data will be reconstructed into the following 35 temporal frames: 12 frames x 10 sec, 8 frames x 30 sec, 6 frames x 90 sec and 9 frames x 300 sec.

Heart rate and blood pressure measurements

During the study day we will monitor heart rate and blood pressure using a cuff connected to a digital blood pressure device at three set time points (at the start of the study day, before administration of salbutamol and after PET/CT scan). In addition we will monitor heartrate and -rhythm during PET/CT scan using a 3-lead ECG connected to a bedside patient monitor (Philips Intellivue MP5).

7.4 Withdrawal of individual subjects

Subjects can leave the study at any time for any reason if they wish to do so without any consequences. The investigator can decide to withdraw a subject from the study for urgent medical reasons.

7.5 Replacement of individual subjects after withdrawal

When a subject leaves the study, the investigator will try finding a replacement in order to maintain the initial population size. The newly recruited subject must comply with the established inclusion- and exclusion criteria.

7.6 Follow-up of subjects withdrawn from treatment

After a subject's withdrawal, he will be followed at the KRIG by the responsible investigator as frequently as necessary. If necessary, another specialist will be consulted, or the patient will be referred to a specialist for further treatment or investigation.

7.7 Premature termination of the study

We assume it would be unlikely that the study will be terminated prematurely due to the study method. Subjects will undergo a screening to minimize most severe side-effects known from IV salbutamol and propranolol. During the study days vital signs and potassium will be controlled at several set time points. This method is performed previously in the Centre for Human Drug Research (CHDR) without any severe side effects or complications [28]. In the unlikely case we do have to terminate our study prematurely we will perform data analysis on the subjects we have included.

8. SAFETY REPORTING

8.1 Temporary halt for reasons of subject safety

In accordance to section 10, subsection 4, of the WMO, the sponsor will suspend the study if there is sufficient ground that continuation of the study will jeopardize subject health or safety. The sponsor will notify the accredited METC without undue delay of a temporary halt including the reason for such an action. The study will be suspended pending a further positive decision by the accredited METC. The investigator will take care that all subjects are kept informed.

8.2 AEs, SAEs and SUSARs

8.2.1 Adverse events (AEs)

Adverse events are defined as any undesirable experience occurring to a subject during the study, whether or not considered related to the investigational product, trial procedure or the experimental intervention. All adverse events reported spontaneously by the subject or observed by the investigator or his staff will be recorded.

8.2.2 Serious adverse events (SAEs)

A serious adverse event is any untoward medical occurrence or effect that:

- results in death;
- is life threatening (at the time of the event);
- requires hospitalization or prolongation of existing inpatients' hospitalization;
- results in persistent or significant disability or incapacity;
- is a congenital anomaly or birth defect; or
- any other important medical event that did not result in any of the outcomes listed above due to medical or surgical intervention but could have been based upon appropriate judgement by the investigator.

An elective hospital admission will not be considered as a serious adverse event.

The investigator will report all SAEs to the sponsor without undue delay after obtaining knowledge of the events. The sponsor will report the SAEs through the web portal *ToetsingOnline* to the accredited METC that approved the protocol, within 7 days of first knowledge for SAEs that result in death or are life threatening followed by a period of maximum of 8 days to complete the initial preliminary report.

All other SAEs will be reported within a period of maximum 15 days after the sponsor has first knowledge of the serious adverse events.

8.2.3 Suspected unexpected serious adverse reactions (SUSARs)

Adverse reactions are all untoward and unintended responses to an investigational product related to any dose administered.

Unexpected adverse reactions are SUSARs if the following three conditions are met:
- the event must be serious (see chapter 9.2.2);
- there must be a certain degree of probability that the event is a harmful and an undesirable reaction to the medicinal product under investigation, regardless of the administered dose;
- the adverse reaction must be unexpected, that is to say, the nature and severity of the adverse reaction are not in agreement with the product information as recorded in: Summary of Product Characteristics (SPC) for an authorized medicinal product; Investigator's Brochure for an unauthorized medicinal product.

The sponsor will report expedited the following SUSARs through the web portal *ToetsingOnline* to the METC:
- SUSARs that have arisen in the clinical trial that was assessed by the METC;
- SUSARs that have arisen in other clinical trials of the same sponsor and with the same medicinal product, and that could have consequences for the safety of the subjects involved in the clinical trial that was assessed by the METC.

The remaining SUSARs are recorded in an overview list (line-listing) that will be submitted once every half year to the METC. This line-listing provides an overview of all SUSARs from the study medicine, accompanied by a brief report highlighting the main points of concern.
The expedited reporting of SUSARs through the web portal Eudravigilance or ToetsingOnline is sufficient as notification to the competent authority.
The sponsor will report expedited all SUSARs to the competent authorities in other Member States, according to the requirements of the Member States.
The expedited reporting will occur not later than 15 days after the sponsor has first knowledge of the adverse reactions. For fatal or life-threatening cases, the term will be maximal 7 days for a preliminary report with another 8 days for completion of the report.

8.3 Annual safety report

In addition to the expedited reporting of SUSARs, the sponsor will submit, once a year throughout the clinical trial, a safety report to the accredited METC, competent authority, and competent authorities of the concerned Member States.

This safety report consists of:

- a list of all suspected (unexpected or expected) serious adverse reactions, along with an aggregated summary table of all reported serious adverse reactions, ordered by organ system, per study;
- a report concerning the safety of the subjects, consisting of a complete safety analysis and an evaluation of the balance between the efficacy and the harmfulness of the medicine under investigation.

8.4 Follow-up of adverse events

All AEs will be followed until they have abated, or until a stable situation has been reached. Depending on the event, follow up may require additional tests or medical procedures as indicated, and/or referral to the general physician or a medical specialist. SAEs need to be reported till end of study within the Netherlands, as defined in the protocol

8.5 Safety Committee

The advice(s) of the safety committee will only be sent to the sponsor of the study. Should the sponsor decide not to fully implement the advice of the safety committee, the sponsor will send the advice to the reviewing METC, including a note to substantiate why (part of) the advice of the safety committee will not be followed.

9. STATISTICAL ANALYSIS

In general, for differences between the effect of salbutamol in combination with placebo, and salbutamol in combination with propranolol either paired t-tests or Wilcoxon signed-rank tests will be used. Statistical analysis will be performed in close collaboration with our local Medical Statistics Department. More specific information on the statistics to be used for the different study parameters can be found below.

9.1 Primary study parameter

Our primary study parameter is glucose uptake by BAT, as measured by dynamic [^{18}F]FDG PET acquisition. Patlak graphical analysis method [43] will be used to determine the fractional extraction of [^{18}F]FDG (Ki) in BAT (http://www.turkupetcentre.net/petanalysis/model_patlak_interpretation.html). Patlak analysis will be based on the plasma input curve (time-radioactivity curve from the aorta), the BAT time-radioactivity curve, and the time in which [^{18}F]FDG has reached steady-state in BAT (approximately 8-10 minutes). Based in the Ki we will calculate metabolic rate of glucose (MRglu).

MRglu = (Glucose concentration in plasma / Lumped constant) * Ki.

(Lumped constant = 1.14 for adipose tissue)

- Differences between the effect of salbutamol in combination with placebo, and salbutamol in combination with propranolol will be assessed by paired-sample t-test or Mann-Whitney test.

9.2 Other study parameters

- Energy expenditure, as measured by indirect calorimetry
    - To compare the effect of salbutamol in combination with placebo with the effect of salbutamol in combination with propranolol paired-sample t-test or Mann-Whitney test will be performed.
- Serum markers for lipid metabolism (triglycerides (TG), total cholesterol (TC), high density lipoprotein cholesterol (HDL-C), low density lipoprotein cholesterol (LDL-C), free fatty acids)
    - To compare the effect of salbutamol in combination with placebo with the effect of salbutamol in combination with propranolol paired-sample t-test or Mann-Whitney test will be performed.
- Lipid pathway analysis (lipidomics)
    - To compare the effect of salbutamol in combination with placebo with the effect of salbutamol in combination with propranolol paired-sample t-test or Mann-Whitney test will be performed, in combination with multiple testing correction via false discovery rate estimation.

- Serum markers for glucose metabolism (glucose, insulin)
  - To compare the effect of salbutamol in combination with placebo with the effect of salbutamol in combination with propranolol paired-sample t-test or Mann-Whitney test will be performed.
- Circulating plasma BAT markers (*e.g.* microRNAs)
  - To compare the effect of salbutamol in combination with placebo with the effect of salbutamol in combination with propranolol paired-sample t-test or Mann-Whitney test will be performed.

10. ETHICAL CONSIDERATIONS

10.1 Regulation statement

The study will be conducted according to the principles of the Declaration of Helsinki (version 8, Fortaleza, Brazil, October 2013) and in accordance with the Medical Research Involving Human Subjects Act (WMO).

10.2 Recruitment and consent

Subjects will be recruited via flyers (see E3), both in the LUMC and in university buildings in Leiden. Hereupon they can approach the researchers via email or telephone. Their eligibility is assessed with a short telephonic interview, after which patient information will be sent by regular mail or email including the informed consent form (see document E1/E2) and flyer 'Medisch-wetenschappelijk onderzoek. Algemene informatie voor de proefpersoon'. After one week of consideration about their decision a screening appointment will be planned. The screening appointment starts with discussing the study information and informed consent form once again in person with the researcher. Thereafter the informed consent form will be signed by the participant and the researcher.

10.3 Benefits and risks assessment, group relatedness

In this study only young healthy male subjects will be included. They are considered able to reasonably judge about their participation in this study after being thoroughly informed by the researcher about the risks and burden of the study. We will not include incapacitated subjects or minors. Subjects will not directly benefit from participation in this study. However, the results from this study are indispensable for unravelling the working mechanism of human BAT. In addition, the results from this study could reveal new therapeutic targets to activate human BAT and could therefore contribute to the fight against the worldwide obesity epidemic. Therefore, the risks of this study are considered negligible. Furthermore, the burden of this study is minimalized using several precautions, which can be found in paragraph 6 'Investigational product' and in paragraph 13 'Structural risk analysis'.

10.4 Compensation for injury

The sponsor/investigator has a liability insurance which is in accordance with article 7 of the WMO.

The sponsor (also) has an insurance which is in accordance with the legal requirements in the Netherlands (Article 7 WMO). This insurance provides cover for damage to research subjects through injury or death caused by the study.

The insurance applies to the damage that becomes apparent during the study or within 4 years after the end of the study.

10.5 Incentives (if applicable)

Subjects will be paid € 100, - for participation in the study. In addition, travel expenses will be repaid, € 0.19/km if subjects travel by car. Public transport will be recompensed on basis of second class.

11. ADMINISTRATIVE ASPECTS, MONITORING AND PUBLICATION

11.1 Handling and storage of data and documents

At the start of the study, subjects will be assigned a random number that will not change during the study. This number is linked with the name, e-mail address, date of birth, and telephone number of the subject in a password protected file. All data and material will be coded only with this number, no identifiable details will be used. Only members of the research team can access the protected file that links the number with subject details. Handling of personal data complies with the General Data Protection Regulation (GDPR).

Storage of data

All raw PET/CT data files will be stored at the Department of Radiology coded by the individual patient study ID. All other data will be collected and stored in a database that complies with all regulations (Castor). From the moment of signing the informed consent during the screening visit the subject will be enrolled in Castor. Data will be stored for 15 years. Subjects that do not agree with this are excluded from participation in the study. The participant can withdraw his informed consent at any time, without having to give a reason. Data already obtained until that moment will still be used. However, data will not be stored nor used for future research. Body material will be destroyed. Data obtained until that moment will be archived for 15 years.

Information about unexpected findings

In case of an unexpected finding during this study the researcher will inform the subject. The subject can inform his general practitioner or medical specialist for further treatment or evaluation. Approval for this procedure is given in the informed consent.

More information about data protection

The responsible person for data handling is the principal researcher of this study, Dr. M.R. Boon. Contact details are shared with the subjects in the patient information form. In case of questions and complains about the procedures used for storage and handling of data and documents subjects can contact the research location. Furthermore, contact details of the ‘Functionaris voor de Gegevensbescherming’ of the LUMC are included in the patient information folder (infoavg@lumc.nl).

11.2 Monitoring and Quality Assurance

Monitoring will be executed by (internal) monitors of the LUMC according to the monitor plan.

11.3 Amendments

A ‘substantial amendment’ is defined as an amendment to the terms of the METC application, or to the protocol or any other supporting documentation, that is likely to affect to a significant degree:

- the safety or physical or mental integrity of the subjects of the trial;
- the scientific value of the trial;
- the conduct or management of the trial; or
- the quality or safety of any intervention used in the trial.

All substantial amendments will be notified to the METC and to the competent authority. Non-substantial amendments will not be notified to the accredited METC and the competent authority but will be recorded and filed by the sponsor.

11.4 Annual progress report

The sponsor/investigator will submit a summary of the progress of the trial to the accredited METC once a year. Information will be provided on the date of inclusion of the first subject, numbers of subjects included and numbers of subjects that have completed the trial, serious adverse events/ serious adverse reactions, other problems, and amendments.

11.5 Temporary halt and (prematurely) end of study report

The sponsor will notify the accredited METC and the competent authority of the end of the study within a period of 90 days. The end of the study is defined as the last patient’s last visit. The sponsor will notify the METC immediately of a temporary halt of the study, including the reason of such an action. In case the study is ended prematurely, the sponsor will notify the accredited METC and the competent authority within 15 days, including the reasons for the premature termination. Within one year after the end of the study, the investigator/sponsor will submit a final study report with the results of the study, including any publications/abstracts of the study, to the accredited METC and the Competent Authority.

11.6 Public disclosure and publication policy

The results of this study will be disclosed unreservedly in scientific journals. Publication will be performed according the principles of the CCMO-statement on publication policy, date March 2002 (https://www.ccmo.nl/publicaties/publicaties/2002/03/15/ccmo-notitie-publicatiebeleid).

12. STRUCTURED RISK ANALYSIS

The investigational product in this study is 250 µg salbutamol, which will be given via IV administration with the aim to activate human BAT. Risks concerning the non-investigational

product propranolol are considered negligible, as there is experience in the department of Radiology with the use of propranolol, at the dosage that we propose, to suppress human BAT before performing an [^{18}F]FDG PET/CT. In addition, risks concerning the [^{18}F]FDG PET/CT scan mainly consist of radiation burden. In this study radiation burden will reach a total of 8.4 mSv, which is below the threshold of 10 mSv (**Appendix A**). PET/CT scans are employed in the LUMC for multiple years and will be performed by qualified personnel from the radiology department.
We will elaborate this risk analysis on the IV administration of 250 µg salbutamol.

12.1 Potential issues of concern

a. Level of knowledge about mechanism of action

Salbutamol is approved for clinical care since 1968 for the prevention and treatment of bronchospasms in asthma and COPD. There is thus a very good level of understanding about the mechanism of action from both non-clinical as clinical studies. Salbutamol works selectively on β2-ARs, which are amongst others present on bronchial muscles. However, β2-ARs are also present on adipose tissue and salbutamol is shown to induce increased lipolysis and lipid oxidation after IV administration[13-15].

b. Previous exposure of human being with the test product

For detailed information about the mechanism of action of salbutamol in human we refer to the ‘Summary of Product Characteristics’ (SPC), which can be found on the website of the ‘College ter Beoordeling van Geneesmiddelen’ (CBG) (https://www.geneesmiddeleninformatiebank.nl/nl/, registration number RVG 07686).

c. Can the primary or secondary mechanism be induced in animals and/or in ex-vivo human cell material?

Pharmacological stimulation and inhibition of the ADRB2 on human brown adipocytes in an *in vitro* setting has previously confirmed that activation of BAT, as determined by increased lipolysis and thermogenesis, is generated through the ADRB2[1].

d. Selectivity of the mechanism to target tissue in animals and/or human beings

The ADRB2 is a G-protein-coupled receptor and is widely distributed through several organs systems in the human body [44]. The ADRB2 is most abundantly found on smooth muscle cells in the pulmonary tract, where it causes muscle relaxation and bronchodilatation. Therefore, ADRB2 agonists are indicated for the symptomatic treatment of the pulmonary obstructive diseases asthma and COPD. In the cardiovascular system, the ADRB2 is also present, however the ADRB1 is dominant over the ADRB2. Stimulation

of these adrenergic receptors causes an increased inotropic and chronotropic effect [44]. The third main organ system influenced by beta-adrenergic receptors is the metabolic system. More specifically and relevant for this study, it is recently shown that human BAT shows a high proportion of ADRB2 mRNA[1].

e. Analysis of potential effect

After intravenous administration, salbutamol has a rather large volume of distribution, indicating extensive uptake from the blood stream to tissues [45]. We are interested in the effect of salbutamol on BAT and therefore will administer the drug systemically, predicting a sufficient drug level on effect-site. Salbutamol is approved for clinical care in the form of an IV injection in a dosage of 250 µg. There is currently no knowledge about the drug level needed for an effect on adipose tissue. Previous studies have investigated the effect of salbutamol on metabolism using a continuous infusion of salbutamol. They found increased energy expenditure and increased plasma free fatty acids and glycerol levels, indicating increased lipolysis, after salbutamol infusion ranging from 0.05-0.1 µg/kg/min salbutamol for 45 minutes in normal-weight to moderate-obese participants [13-15]. Altogether, we consider a single IV injection in a dosage of 250 µg to be safe, since this is the recommended dosage for clinical use, and effective, when taking previous studies in thought.

f. Pharmacokinetic considerations

Elimination route of salbutamol goes predominantly via kidneys in unchanged form. Plasma half-life is 4-6 hours. For pharmacokinetic considerations we refer to the 'Summary of Product Characteristics' (SPC), which can be found on the website of the 'College ter Beoordeling van Geneesmiddelen' (CBG) (https://www.geneesmiddeleninformatiebank.nl/nl/, registration number RVG 07686) and to Morgan et al. Br. J. Clin. Pharmac. (1986) [45].

g. Study population

Study subjects are lean (BMI ≥ 18 and ≤ 25 kg/m^2), healthy males with an age between 18-35 years old.

h. Interaction with other products

Salbutamol will be given in combination with either placebo or 80 mg propranolol capsules. No interaction between the two products is described. Propranolol is a beta-blocker and thus antagonizes the effect of salbutamol, which is the proposed effect in this study.

i. Predictability of effect

Our primary study parameter is glucose uptake by BAT, as measured by dynamic [^{18}F]FDG PET acquisition. We expect increased glucose uptake by BAT as a measure of BAT activation after stimulation of the β2-AR. In addition, we expect increased levels of free fatty acids and glycerol, as a marker of increased lipolysis in adipose tissue, and increased resting energy expenditure, as an indicator of the effect on whole body metabolism.

j. Can effects be managed?

Salbutamol will be administered systemically in a single dose over the time course of 5 minutes. Several pre-cautions will be undertaken to prevent and monitor possible side effects, as previously discussed in paragraph 6 ‘Investigational product’. Table 4 provides an overview of the precautions we will be undertaking regarding the side effects of concern. Taking these pre-cautions into account, we do not expect severe adverse effects during this study.

Table 4 Side effects of concern and the to be undertaken precautions.
BP=Blood pressure; CV=cardiovascular; HR=heart rate.

Possible side effects	Precautions	Moment
**Cardiovascular (CV) side effects**	ECG<br>Medical CV history<br>Family CV history	Screening visit
	Monitoring HR + BP	- Start study day<br>- Before salbutamol administration<br>- After PET/CT scan
	Continuous HR monitoring	During PET/CT scan
**Hypokalemia**	Medication use	Screening
	Serum potassium level monitoring	Study day (before and after salbutamol administration)
**Lactic acidosis**	Monitoring any signs of dyspnea	Study day

12.2 Synthesis

Salbutamol IV injection is approved for clinical care in a dosage of 250 µg and we will not exceed this dosage. At all times there will be a medical doctor and nurse present during the study days. In addition, we will be undertaking several precautions to prevent and minimize risk after administration (Table 4). This protocol with the same precautions is previously performed by Kruizinga et al. [28] in the Centre for Human Drug Research (CHDR) in Leiden. They did not report any severe side effects, neither when using a double dosage of 500 µg IV injection of salbutamol. Taken together, we consider a minimal risk of the current study.

Appendix A. Verantwoording stralingsbelasting

Stralingsbelasting bij dynamische ^{18}F-FDG PET/CT scans ten behoeve van medisch ethische toetsing van het onderzoekprotocol *"Stimulation of the beta-2 adrenergic receptor for activating human brown adipose tissue".*

Bij het onderzoek worden twee dynamische ^{18}F-FDG scans van gezonde vrijwilligers van de supraclaviculaire regio gemaakt. De stralingsbelasting (uitgedrukt als effectieve dosis) van degene die participeert in het onderzoek wordt geschat op ongeveer 8,4 mSv. Dit is de berekende stralingsbelasting van 185 MBq ^{18}F-FDG met een conversiefactor van 1,9 x 10^{-2} mSv/MBq [1] (3,5 mSv per scan) en een low-dose CT-scan, inclusief surview (30 mA_{eff}, 120 kVp) (0,7 mSv per scan).

De Nederlandse Commissie voor Stralingsdosimetrie geeft richtlijnen voor blootstellingen van vrijwilligers bij wetenschappelijk onderzoek [2]. Deze richtlijnen gaan uit van vijf categorieën voor de effectieve dosis. Voor volwassen vrijwilligers (mannen tussen de 30 en 39 jaar) worden de volgende categorieën onderscheiden:

Categorie I	*: < 0,1 mSv*
Categorie IIa	*: 0,1 – 1,0 mSv*
Categorie IIb	*: 1,0 – 10,0 mSv*
Categorie IIIa	*: 10,0 – 20,0 mSv*
Categorie IIIb	*: >20,0 mSv*

De toetsing van de stralingsbelasting van vrijwilligers bij biomedisch onderzoek dient te worden getoetst aan de volgende criteria:

I (< 0,1 mSv) triviaal risico op schade na blootstelling aan straling (≤ 5 op een miljoen), er moet worden aangetoond dat het onderzoek tenminste tot doel heeft de kennis te vergroten

IIa (0,1-1,0 mSv) risico in de orde van grootte van 5 op 100.000, het onderzoek moet tenminste leiden tot gezondheidsvoordelen voor toekomstige patiënten

IIb (1,0-10,0 mSv) risico in de orde van grootte van 5 op 10.000, het betreft onderzoek dat gericht is op de diagnose, behandeling of preventie van de ziekte

IIIa (10,0-20,0 mSv) risico in de orde van grootte van maximaal 1 op de 1.000, het onderzoek levert een substantiële bijdrage aan behandeling en preventie van ernstige aandoeningen

IIIb (> 20,0 mSv) risico ≥ 1 op 1.000, het onderzoek moet direct gerelateerd zijn aan het redden van levens of het verzachten van ernstige aandoeningen

Het onderzoek valt binnen de criteria van categorie IIb, dit betekent dat het voorgestelde onderzoek voor hen getoetst moet worden aan het criterium '*het betreft onderzoek dat gericht is op de diagnose, behandeling of preventie van de ziekte'.*

Referenties:

1. *ICRP. Radiation dose to patients from radiopharmaceuticals. Addendum 3 to ICRP Publication 53. ICRP Publication 106. Approved by the Commission in October 2007. Ann ICRP. 2008;38(1-2):1–197*
2. *Nederlandse Commissie voor Stralingsdosimetrie, Human Exposure to Ionising Radiation for Clinical and Research Purposes: Radiation Dose & Risk Estimates. NCS 26. Mei 2016.*

Dr. F.H.P. van Velden (klinisch fysicus), 6 juli 2020
Afdeling Radiologie, Sectie Nucleaire Geneeskunde, LUMC, tel 071-526 5726

13. REFERENCES

1. Blondin, D.P., et al., *Human Brown Adipocyte Thermogenesis Is Driven by β2-AR Stimulation.* Cell Metab, 2020. **32**(2): p. 287-300.e7.
2. Pi-Sunyer, F.X., *The obesity epidemic: pathophysiology and consequences of obesity.* Obes Res, 2002. **10 Suppl 2**: p. 97s-104s.
3. Ruiz, J.R., et al., *Role of Human Brown Fat in Obesity, Metabolism and Cardiovascular Disease: Strategies to Turn Up the Heat.* Prog Cardiovasc Dis, 2018. **61**(2): p. 232-245.
4. Cannon, B. and J. Nedergaard, *Brown adipose tissue: function and physiological significance.* Physiol Rev, 2004. **84**(1): p. 277-359.
5. Khedoe, P.P., et al., *Brown adipose tissue takes up plasma triglycerides mostly after lipolysis.* J Lipid Res, 2015. **56**(1): p. 51-9.
6. Bartness, T.J., C.H. Vaughan, and C.K. Song, *Sympathetic and sensory innervation of brown adipose tissue.* Int J Obes (Lond), 2010. **34 Suppl 1**(0 1): p. S36-42.
7. Kooijman, S., J.K. van den Heuvel, and P.C.N. Rensen, *Neuronal Control of Brown Fat Activity.* Trends in Endocrinology & Metabolism, 2015. **26**(11): p. 657-668.
8. Berbée, J.F., et al., *Brown fat activation reduces hypercholesterolaemia and protects from atherosclerosis development.* Nat Commun, 2015. **6**: p. 6356.
9. Yoshida, T., et al., *Anti-obesity and anti-diabetic effects of CL 316,243, a highly specific beta 3-adrenoceptor agonist, in yellow KK mice.* Life Sci, 1994. **54**(7): p. 491-8.
10. Bengtsson, T., B. Cannon, and J. Nedergaard, *Differential adrenergic regulation of the gene expression of the beta-adrenoceptor subtypes beta1, beta2 and beta3 in brown adipocytes.* Biochem J, 2000. **347 Pt 3**(Pt 3): p. 643-51.
11. Cypess, A.M., et al., *Activation of human brown adipose tissue by a β3-adrenergic receptor agonist.* Cell Metab, 2015. **21**(1): p. 33-8.
12. Wachter, S.B. and E.M. Gilbert, *Beta-adrenergic receptors, from their discovery and characterization through their manipulation to beneficial clinical application.* Cardiology, 2012. **122**(2): p. 104-12.
13. Schiffelers, S.L., et al., *beta(1)- and beta(2)-Adrenoceptor-mediated thermogenesis and lipid utilization in obese and lean men.* J Clin Endocrinol Metab, 2001. **86**(5): p. 2191-9.
14. Hoeks, J., et al., *Effect of beta1- and beta2-adrenergic stimulation on energy expenditure, substrate oxidation, and UCP3 expression in humans.* Am J Physiol Endocrinol Metab, 2003. **285**(4): p. E775-82.
15. Oomen, J.M., et al., *β2-Adrenergic Receptor Polymorphisms and Salbutamol-Stimulated Energy Expenditure.* The Journal of Clinical Endocrinology & Metabolism, 2005. **90**(4): p. 2301-2307.
16. WHO. *Obesity and Overweight*. 2020 [cited 2020 June 10 2020]; Available from: https://www.who.int/news-room/fact-sheets/detail/obesity-and-overweight.
17. Ouellet, V., et al., *Brown adipose tissue oxidative metabolism contributes to energy expenditure during acute cold exposure in humans.* The Journal of Clinical Investigation, 2012. **122**(2): p. 545-552.
18. Yoneshiro, T., et al., *Recruited brown adipose tissue as an antiobesity agent in humans.* J Clin Invest, 2013. **123**(8): p. 3404-8.
19. van der Lans, A.A., et al., *Cold acclimation recruits human brown fat and increases nonshivering thermogenesis.* J Clin Invest, 2013. **123**(8): p. 3395-403.
20. Baskin, A.S., et al., *Regulation of Human Adipose Tissue Activation, Gallbladder Size, and Bile Acid Metabolism by a β3-Adrenergic Receptor Agonist.* Diabetes, 2018. **67**(10): p. 2113-2125.
21. Carpentier, A.C., et al., *Brown Adipose Tissue Energy Metabolism in Humans.* Front Endocrinol (Lausanne), 2018. **9**: p. 447.
22. Richard, M.A., H. Pallubinsky, and D.P. Blondin, *Functional characterization of human brown adipose tissue metabolism.* Biochem J, 2020. **477**(7): p. 1261-1286.

23. Nahon, K.J., et al., *The effect of mirabegron on energy expenditure and brown adipose tissue in healthy lean South Asian and Europid men.* Diabetes Obes Metab, 2020.
24. Bakker, L.E., et al., *Brown adipose tissue volume in healthy lean south Asian adults compared with white Caucasians: a prospective, case-controlled observational study.* Lancet Diabetes Endocrinol, 2014. **2**(3): p. 210-7.
25. Orava, J., et al., *Different metabolic responses of human brown adipose tissue to activation by cold and insulin.* Cell Metab, 2011. **14**(2): p. 272-9.
26. *Zorginstituut Nederland. Farmacotherapeutisch Kompas. Salbutamol parenteraal.* [cited 2020 16-06]; Available from: https://www.farmacotherapeutischkompas.nl/bladeren/preparaatteksten/s/salbutamol__parenteraal.
27. *Zorginstituut Nederland. Farmacotherapeutisch Kompas.* [cited 2020 16-06]; Available from: https://www.farmacotherapeutischkompas.nl/bladeren/preparaatteksten/s/salbutamol__parenteraal.
28. Kruizinga, M.D., et al., *Pharmacokinetics of intravenous and inhaled salbutamol and tobramycin: An exploratory study to investigate the potential of exhaled breath condensate as a matrix for pharmacokinetic analysis.* Br J Clin Pharmacol, 2020. **86**(1): p. 175-181.
29. Ernande, L., et al., *Relationship of brown adipose tissue perfusion and function: a study through β2-adrenoreceptor stimulation.* J Appl Physiol (1985), 2016. **120**(8): p. 825-32.
30. Mahú, I., et al., *Brain-Sparing Sympathofacilitators Mitigate Obesity without Adverse Cardiovascular Effects.* Cell Metab, 2020. **31**(6): p. 1120-1135.e7.
31. Blaak, E.E., et al., *Role of alpha- and beta-adrenoceptors in sympathetically mediated thermogenesis.* Am J Physiol, 1993. **264**(1 Pt 1): p. E11-7.
32. van Baak, M.A., *The peripheral sympathetic nervous system in human obesity.* Obes Rev, 2001. **2**(1): p. 3-14.
33. Bohn, D., et al., *Intravenous salbutamol in the treatment of status asthmaticus in children.* Crit Care Med, 1984. **12**(10): p. 892-6.
34. Liedtke, A.G., et al., *Selective ß2-Adrenoceptor Agonists and Relevant Hyperlactatemia: Systematic Review and Meta-Analysis.* J Clin Med, 2019. **9**(1).
35. Moore, A., et al., *Pharmacokinetics of Salbutamol Delivered from the Unit Dose Dry Powder Inhaler: Comparison with the Metered Dose Inhaler and Diskus Dry Powder Inhaler.* J Aerosol Med Pulm Drug Deliv, 2017. **30**(3): p. 164-172.
36. *Zorginstituut Nederland. Farmacotherapeutisch Kompas. Propranolol.* [cited 2020 25-06]; Available from: https://www.farmacotherapeutischkompas.nl/bladeren/preparaatteksten/p/propranolol__cardiovasculair_of_neurologisch_.
37. George, A., et al., *Pilot study of propranolol premedication to reduce FDG uptake in brown adipose tissue on PET scans of adolescent and young adult oncology patients.* Pediatr Hematol Oncol, 2017. **34**(3): p. 149-156.
38. Agrawal, A., N. Nair, and N.S. Baghel, *A novel approach for reduction of brown fat uptake on FDG PET.* Br J Radiol, 2009. **82**(980): p. 626-31.
39. Parysow, O., et al., *Low-dose oral propranolol could reduce brown adipose tissue F-18 FDG uptake in patients undergoing PET scans.* Clin Nucl Med, 2007. **32**(5): p. 351-7.
40. Söderlund, V., S.A. Larsson, and H. Jacobsson, *Reduction of FDG uptake in brown adipose tissue in clinical patients by a single dose of propranolol.* Eur J Nucl Med Mol Imaging, 2007. **34**(7): p. 1018-22.
41. Boellaard, R., et al., *FDG PET/CT: EANM procedure guidelines for tumour imaging: version 2.0.* Eur J Nucl Med Mol Imaging, 2015. **42**(2): p. 328-54.
42. Sanchez-Delgado, G., et al., *Reliability of resting metabolic rate measurements in young adults: Impact of methods for data analysis.* Clin Nutr, 2018. **37**(5): p. 1618-1624.

43. Ménard, S.L., et al., *Abnormal in vivo myocardial energy substrate uptake in diet-induced type 2 diabetic cardiomyopathy in rats.* Am J Physiol Endocrinol Metab, 2010. **298**(5): p. E1049-57.
44. Liggett, S.B., *Pharmacogenetics of beta-1- and beta-2-adrenergic receptors.* Pharmacology, 2000. **61**(3): p. 167-73.
45. Morgan, D.J., et al., *Pharmacokinetics of intravenous and oral salbutamol and its sulphate conjugate.* Br J Clin Pharmacol, 1986. **22**(5): p. 587-93.
